# *S*-nitrosylation of the Peroxiredoxin-2 promotes *S*-nitrosoglutathione-mediated lung cancer cells apoptosis via AMPK-SIRT1 pathway

**DOI:** 10.1038/s41419-019-1561-x

**Published:** 2019-04-15

**Authors:** Yihan Zhang, Changning Sun, Guokai Xiao, Hui Shan, Luyao Tang, Yujiao Yi, Wengong Yu, Yuchao Gu

**Affiliations:** 1 Key Laboratory of Marine Drugs, Ministry of Education, School of Medicine and Pharmacy, Ocean University of China, 5 Yushan Road, Qingdao, 266003 China; 2 Laboratory for Marine Drugs and Bioproducts of Qingdao National Laboratory for Marine Science and Technology, Qingdao, 266200 China; 3 Key Laboratory of Glycoscience & Glycotechnology of Shandong Province, Qingdao, 266003 China

## Abstract

Protein *S*-nitrosylation, the redox-based posttranslational modification of a cysteine thiol by the attachment of a nitric oxide (NO) group, is responsible for a variety of signaling effects. Dysregulation of *S*-nitrosylation may be directly linked to cancer apoptotic resistance and cancer therapy outcomes, emphasizing the importance of *S*-nitrosylation in cancer. Peroxiredoxin-2 (Prdx2), an antioxidant enzyme, plays an important role in the protection of cancer cells from oxidative radical damage caused by hydrogen dioxide (H_2_O_2_), which is a potential target for cancer therapy. Our studies showed that, as an endogenous NO carrier, *S*-nitrosoglutathione (GSNO) induced apoptosis in lung cancer cells via nitrosylating Prdx2. The nitrosylation of Prdx2 at Cys51 and Cys172 sites disrupted the formation of Prdx2 dimer and repressed the Prdx2 antioxidant activity, causing the accumulation of endogenous H_2_O_2_. H_2_O_2_ activated AMPK, which then phosphorylated SIRT1 and inhibited its deacetylation activity toward p53 in A549 cells or FOXO1 in NCI-H1299 cells. Taken together, our results elucidate the roles and mechanisms of Prdx2 *S*-nitrosylation at Cys51 and Cys172 sites in lung cancer cells apoptosis and this finding provides an effective lung cancer treatment strategy for managing aberrant Prdx2 activity in lung cancers.

## Introduction

Lung cancer is one of the most common types of malignancies worldwide with a 5-year survival rate of <15%. The majority of patients are diagnosed with an incurable advanced/metastatic stage disease^1,2^. To circumvent resistance to apoptosis is a novel strategy for treating tumors^3^. Therefore, a much deeper understanding of key molecular events involving in apoptotic resistance may lead to better diagnosis and treatment.

Protein *S*-nitrosylation is the posttranslational modification of a cysteine by attaching a nitric oxide (NO) group to major classes of proteins^4^. It modulates the function of target proteins through conformational changes, alteration of protein activity, or regulation of protein–protein interactions^5^. Increasing studies have shown that *S*-nitrosylation status may be directly linked to cancer onset, progression, and treatment resistance^6^, highlighting the possibility to develop *S*-nitrosylation-related anti-cancer therapeutic drugs. As the storage and transport form of NO, *S*-nitrosoglutathione (GSNO) has been proposed to regulate circulating levels of NO and NO-derived species^7^. By transferring its NO moiety to some proteins (*trans*-nitrosylation)^8^, GSNO plays a crucial role of protein *S*-nitrosylation in both physiological and pathological processes^9^. Besides, GSNO can also protect against oxidative stress in the endothelium, myocardium, brain tissues, and other cells^10^. It is reported that GSNO inhibits STAT3 phosphorylation through *S*-nitrosylation of STAT3, to increase apoptosis of head and neck squamous cell carcinoma cells^11^. However, whether GSNO can also induce apoptosis in other cancer cells (such as lung cancer cells) remains unclear.

Peroxiredoxins (Prdxs) are thiol-specific antioxidant enzymes, which have a class of six antioxidant enzymes (Prdx1–6) in mammals. They are typically classified by the number of cysteinyl residues directly involved in catalysis as 2-Cys (Prdx1–4), atypical 2-Cys (Prdx5), and 1-Cys (Prdx6)^12^. Prdx2, widely expressed in various tissues and cells, regulates cell proliferation, apoptosis, and differentiation by altering the intracellular hydrogen peroxide (H_2_O_2_) level^13,14^. Excessive H_2_O_2_ induced by abnormal metabolism or anti-tumor drugs leads to damage in cancer cells^15^, which can be inhibited by the overexpression of Prdx2^16^, suggesting it may be possible to downregulate the expression or activity of Prdx2 for inhibiting the growth of lung cancer cells. So far, only adenanthin has been reported to induce the death of hepatocellular carcinoma cells through targeting Prdx1/2^17^. It is demonstrated that nitrosylation affects the status of Prdx2 catalytic cycle, which is involved in cardiomyocyte differentiation of mouse embryonic stem cells induced by GSNO^18^. Therefore, it should be valuable to explore whether GSNO can regulate Prdx2 activity by nitrosylation and further induce the apoptosis of lung cancer cells.

In this study, we investigated the efficacy of GSNO on lung cancer and the related mechanism. We showed that GSNO nitrosylated Prdx2 at Cys51 and Cys172 sites, which are involved in the formation of homodimer, and inhibited its catalytic cycle, subsequently resulting in local H_2_O_2_ accumulation and further increasing the activity of AMPK. Activated AMPK phosphorylated SIRT1 at Thr344 site, which resulted in the loss of SIRT1 deacetylase activity toward p53 or FOXO1 and further induced lung cancer cell apoptosis. These data may provide new insights into the potential target for lung cancer treatment.

## Result

### GSNO induces cell apoptosis in lung cancer cells

**Fig. 1:**
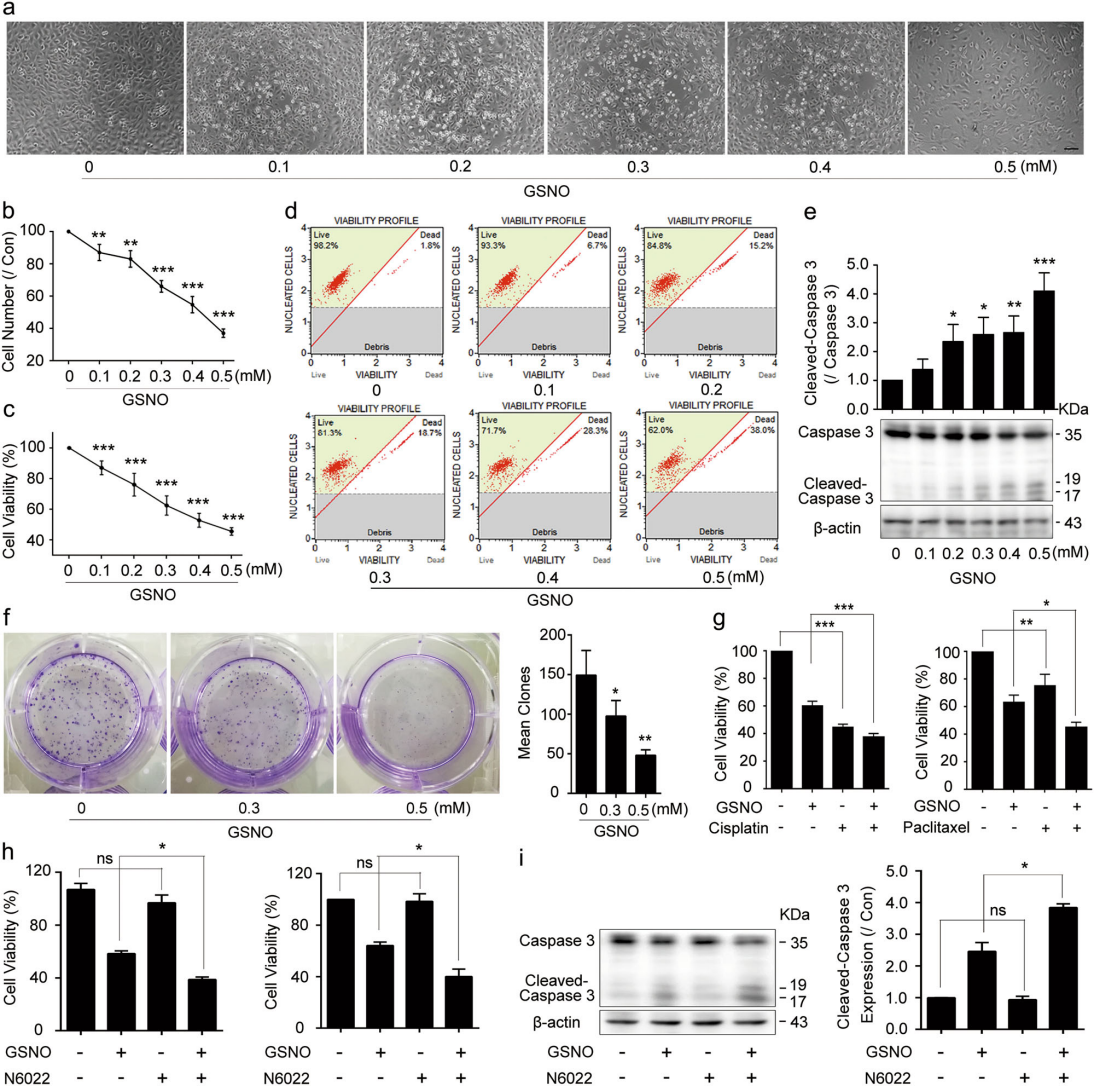
GSNO induces lung cancer cell A549 apoptosis. **a** A549 cells were treated with different concentrations of GSNO (0–0.5 mM) for 24 h (Bar = 20 μm). **b**, **c** Cell number and cell viability analysis of A549 cells after GSNO treatment. **d** Flow cytometry analysis of live cells after treated with GSNO. **e** Expression of Cleavage of Caspase-3 in A549 cells was probed by WB. **f** Colony formation of A549 cells treated with GSNO. **g** The synergistic effects of GSNO (0.3 mM) with cisplatin (10 μM) or paclitaxel (20 μM) on A549 cells. **h** The synergistic effects of GSNO (0.3 mM) with N6022 (0.5 μM) on cell viability analysis of A549 (left) and NCI-H1299 cells (right). **i** Cleavage of Caspase-3 expression in A549 cells treated with GSNO or/and N6022 for 24 h was probed by WB. The data are expressed as the mean ± SD of three independent experiments. *P < 0.05, **P < 0.01, ***P *<* 0.001; ns, not significant, using one-way ANOVA followed by Bonferroni’s multiple comparisons test

To investigate the effect of GSNO on lung cancer cells, we applied it to A549 and NCI-H1299 cells. The results showed that GSNO decreased cell viability and cell numbers in both A549 and NCI-H1299 cells (Fig. 1a–d, Supplementary Fig. 1a–c). Western blotting (WB) analysis further showed that GSNO promoted the activation of Caspase-3 (Fig. 1e, Supplementary Fig. 1d), indicating that GSNO induces cell apoptosis in A549 and NCI-H1299 cells. In clonogenic assays, both A549 and NCI-H1299 cells treated by GSNO formed fewer colonies compared with the control groups (Fig. 1f, Supplementary Fig. 1e). Moreover, combined treatment of GSNO with cisplatin or paclitaxel strongly suppressed cell viability in A549 or NCI-H1299 cells, respectively (Fig. 1g, Supplementary Fig. 1f). Taken together, these results indicate that GSNO inhibits cell growth, survival, and colony formation in lung cancer cells. Interestingly, compared with lung cancer cells, normal lung cells (WI38 and BEAS-2B) were less sensitive to GSNO (Supplementary Fig. 2a). Furthermore, GSNO reductase inhibitor N6022 had synergistic effects with GSNO on cell viability and Caspase-3-mediated apoptosis (Fig. 1h, i). We also treated A549 cells with another NO donor called *S*-Nitroso-*N*-acetylpenicillamine (SNAP). As SNAP produced NO with later decomposition time and lower concentration (Supplementary Fig. 2d), it caused similar but slighter effects of Caspase-3-mediated apoptosis compared with GSNO (Supplementary Fig. 2b–e). Lipopolysaccharides (LPS), which could induce inducible NO synthase^19^ and contribute NO production (Supplementary Fig. 2f), caused similar Caspase-3-mediated apoptosis as GSNO (Supplementary Fig. 2g, h). The above results indicate that GSNO induces cell apoptosis in lung cancer cells through NO production.

### GSNO induces lung cancer cell apoptosis via Prdx2 nitrosylation

**Fig. 2:**
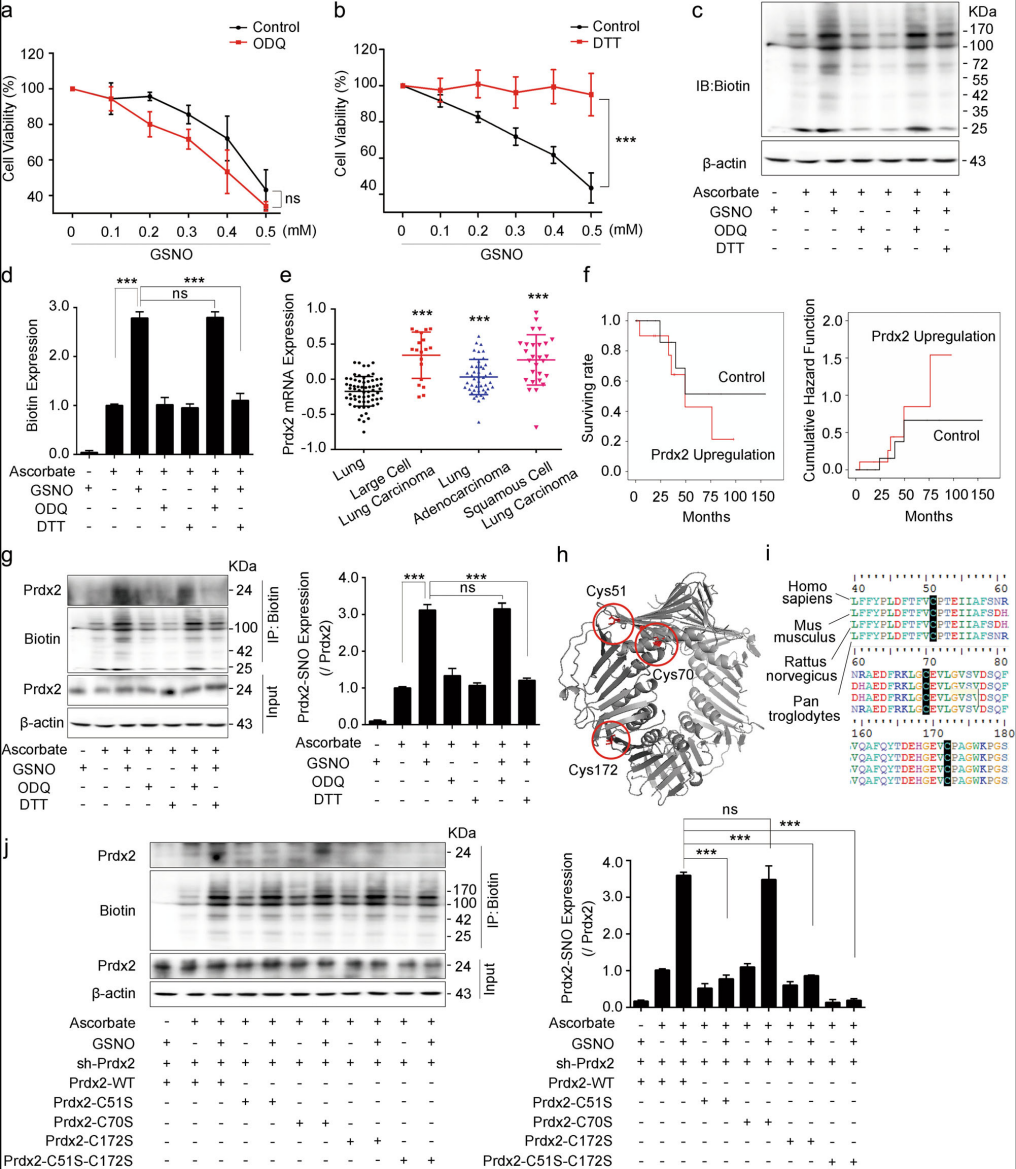
GSNO promotes lung cancer cell apoptosis via Prdx2 nitrosylation. **a** Effect of GSNO on cell viability with or without ODQ (25 μM) for 24 h (ns, not significant, using two-way ANOVA followed by Bonferroni’s multiple comparisons test). **b** Effect of GSNO on cell viability with or without DTT (0.5 mM) for 24 h (***P *<* 0.001, using two-way ANOVA followed by Bonferroni’s multiple comparisons test). **c**, **d** Detection of total protein nitrosylation by biotin-switch assay (***P *<* 0.001; ns, not significant, using one-way ANOVA followed by Bonferroni’s multiple comparisons test). **e** The mRNA expression level of Prdx2 in different kinds of human lung cancer tissues and normal tissues (***P *<* 0.001, using one-way ANOVA followed by Bonferroni’s multiple comparisons test). **f** Kaplan–Meier survival curves for cumulative survival rate and cumulative hazard function of cancer patients according to expression level of Prdx2 mRNA. **g** Detection of Prdx2 nitrosylation by biotin-switch assay (***P *<* 0.001; ns, not significant, using one-way ANOVA followed by Bonferroni’s multiple comparisons test). **h** The 3D structure of Prdx2. The cysteine is shown in red. **i** Sequence alignment of Prdx2 in different species. **j** Expression of Prdx2 nitrosylation with GSNO treatment in different Prdx2 mutantions (***P *<* 0.001; ns, not significant, using one-way ANOVA followed by Bonferroni’s multiple comparisons test). The data are expressed as the mean ± SD of three independent experiments

GSNO exerts its biological function via activating classical sGC/cGMP-dependent pathway or nitrosylating certain proteins. To investigate the potential mechanism of cell apoptosis induced by GSNO, sGC inhibitor 1H-[1,2,4]oxadiazolo[4, 3a]quinoxalin-1-one (ODQ) or nitrosylation scavenger dithiothreitol (DTT) was applied to lung cancer cells. The results showed that ODQ failed to antagonize the effect of GSNO on A549 cells (Fig. 2a, Supplementary Fig. 3a), whereas DTT rescued the decreased cell viability induced by GSNO in A549 and NCI-H1299 cells (Fig. 2b, Supplementary Fig. 3b, c). Moreover, protein nitrosylation was markedly increased upon GSNO exposure and DTT attenuated the nitrosylation while ODQ did not (Fig. 2c, d). These results indicate that GSNO induces cell apoptosis through protein nitrosylation but not in a sGC/cGMP-dependent manner.

To find the potential proteins nitrosylated by GSNO, we focused on Prdxs, which regulate the oxidation status via critical Cys residues^13^. We downloaded publicly available data of 156 human samples (91 lung tumors tissues and 65 normal lung tissues) from GEO (GSE19188)^20^ to assess Prdxs mRNA expression. The results showed that Prdx2 and Prdx4 were expressed higher in large cell lung carcinoma, lung adenocarcinoma, and squamous cell lung carcinoma compared with that in normal lung tissues (Fig. 2e, Supplementary Fig. 3d–h). Our results showed that the mRNA and protein levels of Prdx2 are higher in both A549 and H1299 cells than that in normal lung cells (WI38 and BEAS-2B; Supplementary Fig. 3i, k). However, Prdx4 mRNA level was only increased in NCI-H1299 cells but not in A549 cells (Supplementary Fig. 3j). The analysis of the association between clinicopathological features and high expression of Prdx2 showed that high expression of Prdx2 was significantly correlated with a poorer disease-free survival rate and a higher cumulative hazard function (Fig. 2f). Combined with the finding that GSNO could nitrosylate Prdx2 in mouse embryonic stem cells^18^, we hypothesized Prdx2 nitrosylation might be involved in the process of lung cancer cell apoptosis induced by GSNO. To validate this, we examined Prdx2 expression and its nitrosylation in lung cancer cells after GSNO treatment. The results showed that GSNO treatment did not affect the expression of Prdx2, but significantly increased its nitrosylation, which could be reversed by DTT but not by ODQ (Fig. 2g, Supplementary Fig. 3l). Saville-Griess assay also confirmed that GSNO treatment markedly increased the nitrosylation of both total cellular proteins and Prdx2 (Supplementary Fig. 3m). We further overexpressed Prdx2 in A549 cells and found it could rescue the GSNO-induced cell apoptosis (Supplementary Fig. 3n). Prdx2 contains three cysteine residues (Cys51, Cys70, and Cys172), which are highly conserved within species (Fig. 2h, i). We mutated these Cys sites to Ser separately. After silencing the endogenous Prdx2 by short hairpin RNA (shRNA) strategy, we re-expressed Prdx2 with different mutant forms in A549 cells. Our results showed that the mutation of Cys51 or Cys172, but not Cys70, markedly decreased the nitrosylation level of Prdx2 induced by GSNO (Fig. 2j), which was consistent with previous report that Cys51 and Cys172 were the predominant targets for *S*-nitrosylation on Prdx2^21^.

### GSNO nitrosylates Prdx2 and causes intracellular H_2_O_2_ accumulation

**Fig. 3:**
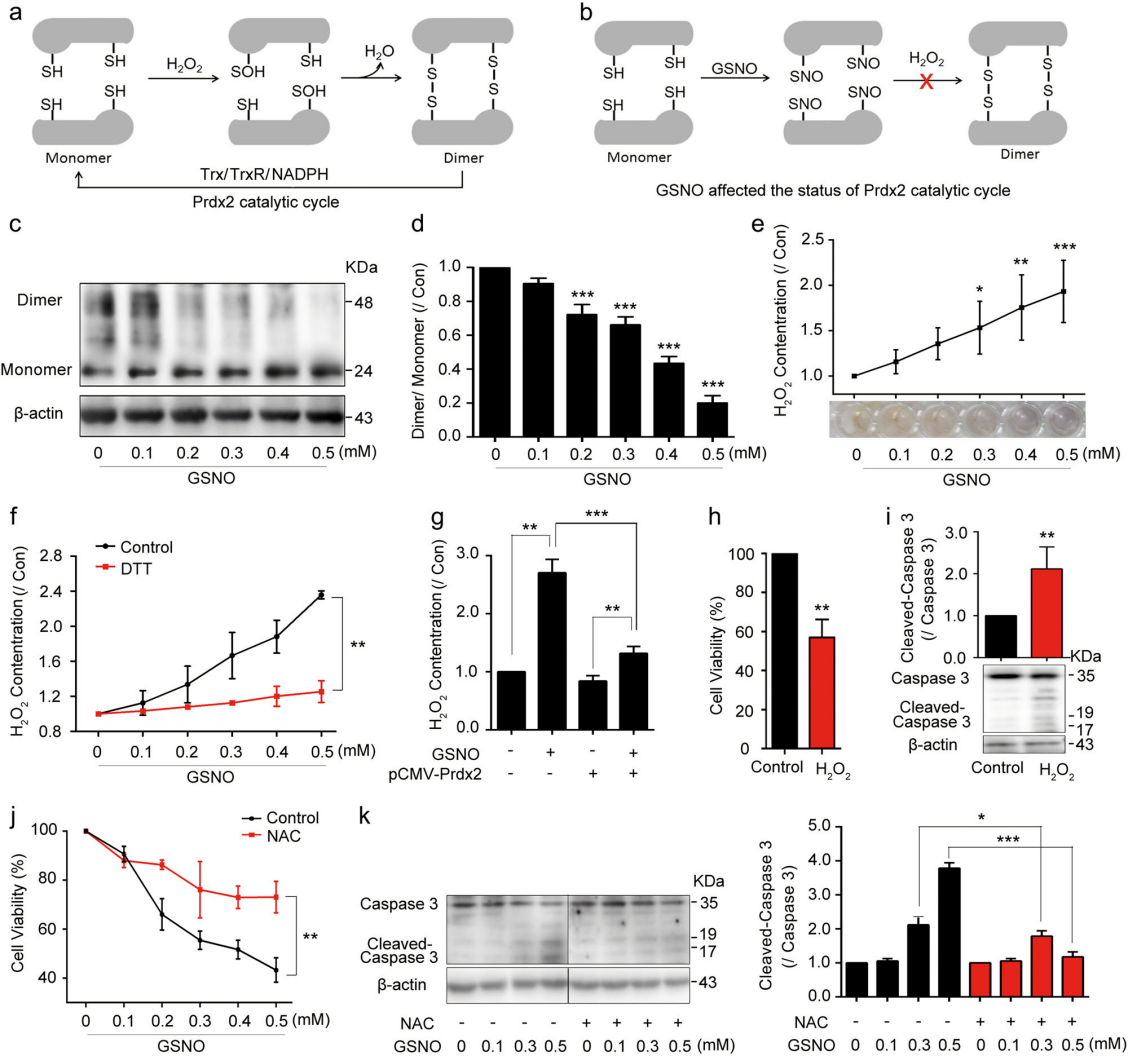
GSNO nitrosylates Prdx2 to inhibit Prdx2 activity and causes H_2_O_2_ accumulation. **a** Schematic representation of physiological Prdx2 catalytic mechanism of Prdx2. **b** GSNO affected the status of Prdx2 catalytic cycle. **c**, **d** Detection of Prdx2 dimmer/monomer status after treating A549 with GSNO for 24 h (***P < 0.001, using one-way ANOVA followed by Bonferroni’s multiple comparisons test). **e** Detection of intracellular H_2_O_2_ after GSNO treatment for 24 h (*P < 0.05, **P < 0.01, ***P < 0.001, using one-way ANOVA followed by Bonferroni’s multiple comparisons test). **f** Detection of intracellular H_2_O_2_ with or without DTT (**P < 0.01, using two-way ANOVA followed by Bonferroni’s multiple comparisons test). **g** Detected intracellular H_2_O_2_ in Prdx2-overexpression cells (**P < 0.01, ***P < 0.001, using one-way ANOVA followed by Bonferroni’s multiple comparisons test). **h** Cell viability analysis of A549 cells with H_2_O_2_ (50 μM) treatment for 24 h (**P *<* 0.01, using unpaired two-tailed Student’s *t*-test). **i** Cleaved Caspase-3 expression in A549 cells with H_2_O_2_ treatment for 24 h (**P *<* 0.01, using unpaired two-tailed Student’s *t*-test). **j** Effect of GSNO on A549 cell viability with or without NAC (2 mM) (**P *<* 0.01, using two-way ANOVA followed by Bonferroni’s multiple comparisons test). **k** Cleaved-Caspase-3 expression in A549 cells with or without NAC (*P *<* 0.05, ***P *<* 0.001, using one-way ANOVA followed by Bonferroni’s multiple comparisons test). The data are expressed as the mean ± SD of three independent experiments

Prdx2 catalytic cycle is related to H_2_O_2_ metabolism^22^. Cys51 of Prdx2 reacts with H_2_O_2_, then with the resolving Cys172 on the second subunit of the dimer to form disulfide bonds^23^ (Fig. 3a). Therefore, the nitrosylation of Prdx2 at Cys51 and Cys172 sites induced by GSNO might inhibit its catalytic cycle, subsequently resulting in H_2_O_2_ accumulation (Fig. 3b). We tested the Prdx2 monomer/dimer ratio and found that GSNO significantly decreased dimer formation (Fig. 3c, d). Consistent with this finding, GSNO increased intracellular H_2_O_2_ accumulation in A549 and NCI-H1299 cells (Fig. 3e, Supplementary Fig. 4a), which could be effectively eliminated by DTT (Fig. 3f). We further overexpressed Prdx2 in A549 cells and found it could reduce H_2_O_2_ accumulation induced by GSNO (Fig. 3g). To mimic the accumulation of H_2_O_2_, H_2_O_2_ was added to the cells. It increased the death of A549 and NCI-H1299 cells (Fig. 3h, i, Supplementary Fig. 4b–h), which is similar to the effect of GSNO. Besides, the apoptosis caused by GSNO was reduced by adding ROS scavenger *N*-acetyl-l-cysteine (NAC, Fig. 3j, k). The above results indicated that GSNO could nitrosylate Prdx2 and inhibit its catalytic cycle, subsequently resulting in intracellular H_2_O_2_ accumulation and inducing lung cancer cell death.

### GSNO induces cell death via activating AMPK

**Fig. 4:**
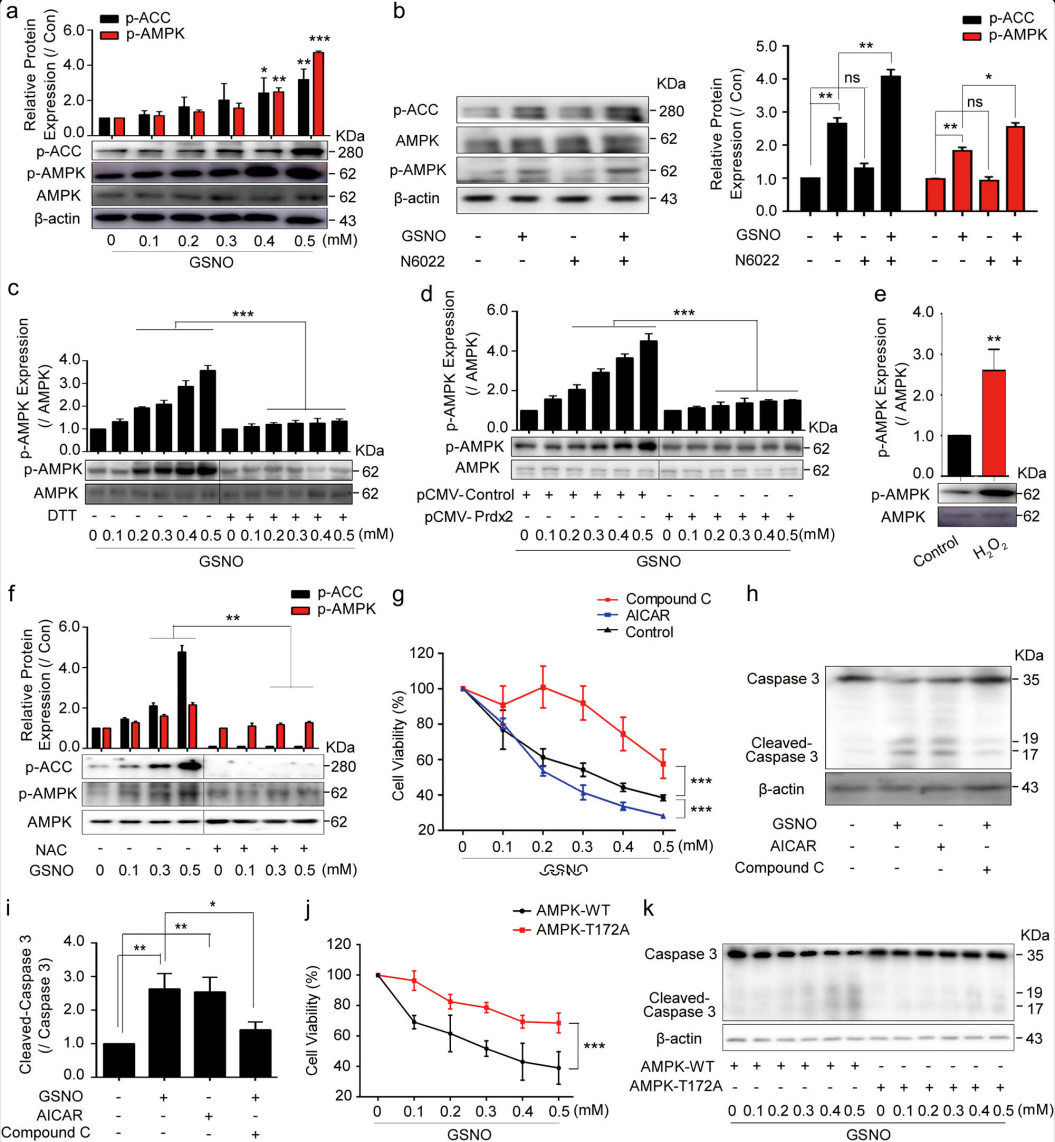
GSNO induces cell death via activating AMPK. **a** Expression of p-ACC and p-AMPK/AMPK after GSNO treatment for 24 h in A549 cells (*P < 0.05, **P < 0.01, ***P < 0.001, using one-way ANOVA followed by Bonferroni’s multiple comparisons test). **b** Effect of N6022 on the expression of p-ACC and p-AMPK/AMPK when treating A549 cells with GSNO (*P *<* 0.05, **P *<* 0.01; ns, not significant, using one-way ANOVA followed by Bonferroni’s multiple comparisons test). **c** DTT attenuated the increased expression of AMPK phosphorylation induced by GSNO in A549 cells (***P *<* 0.001, using two-way ANOVA followed by Bonferroni’s multiple comparisons test). **d** The expression of AMPK phosphorylation in Prdx2-overexpression A549 cells after GSNO treatment for 24 h (***P *<* 0.001, using two-way ANOVA followed by Bonferroni’s multiple comparisons test). **e** Expression of AMPK phosphorylation after H_2_O_2_ (50 μM) treatment for 24 h in A549 cells (**P *<* 0.01, using unpaired two-tailed Student’s *t*-test). **f** The effect of NAC on the expression of AMPK phosphorylation and p-ACC in A549 cells (**P *<* 0.01, using two-way ANOVA followed by Bonferroni’s multiple comparisons test). **g** The effect of GSNO on cell viability with AICAR (1 mM) or Compound C (20 μM) (***P *<* 0.001, using two-way ANOVA followed by Bonferroni’s multiple comparisons test). **h**, **i** The effect of GSNO on Cleavage of Caspase-3 expression with AICAR or Compound C (*P *<* 0.05, **P *<* 0.01, using one-way ANOVA followed by Bonferroni’s multiple comparisons test). **j** The effect of GSNO on cell viability in AMPK-WT and AMPK-T172A-mutant groups (***P *<* 0.001, using two-way ANOVA followed by Bonferroni’s multiple comparisons test). **k** The effect of GSNO on Cleavage of Caspase-3 expression in AMPK-WT and AMPK-T172A-mutant groups. The data are expressed as the mean ± SD of three independent experiments

AMPK is a key energetic sensor and regulator of cellular metabolism that can be activated by the elevation of intracellular H_2_O_2_ levels^24^. We found that GSNO increased the phosphorylation of AMPK and ACC in a concentration-dependent manner in A549 and NCI-H1299 cells (Fig. 4a, Supplementary Fig. 5a). In addition, N6022, SNAP, and LPS had synergistic effects with GSNO, respectively (Fig. 4b; Supplementary Fig. 5b, c). The activated AMPK induced by GSNO could be reversed by the application of DTT or overexpression of Prdx2 (Fig. 4c, d). Moreover, the addition of H_2_O_2_ to A549 and NCI-H1299 cells could mimic the effect of GSNO on the increase of AMPK phosphorylation (Fig. 4e; Supplementary Fig. 5d) and the activation of AMPK caused by GSNO was reduced by adding NAC (Fig. 4f). To confirm whether AMPK activation contributes to GSNO-induced lung cancer cell apoptosis, we used AMPK inhibitor dorsomorphin (Compound C) and AMPK activator 5-aminoimidazole-4-carboxamide 1-β-d-ribofuranoside (AICAR) to regulate the activity of AMPK. As expected, AICAR increased AMPK phosphorylation, whereas Compound C attenuated AMPK phosphorylation caused by GSNO (Supplementary Fig. 5e, f). Moreover, Compound C but not AICAR attenuated the cell damage caused by GSNO in A549 and NCI-H1299 cells (Fig. 4g–i; Supplementary Fig. 5g). As Compound C has been reported exerting some side effects^24^, we transfected alpha-subunit T172A-mutant AMPK into A549 cells. Our results showed that similar to Compound C, cell viability and apoptosis were rescued in T172A-mutant group (Fig. 4j, k, Supplementary Fig. 5j). These results demonstrate that GSNO induces cell death via activating AMPK.

### AMPK phosphorylation inhibits SIRT1 activity

**Fig. 5:**
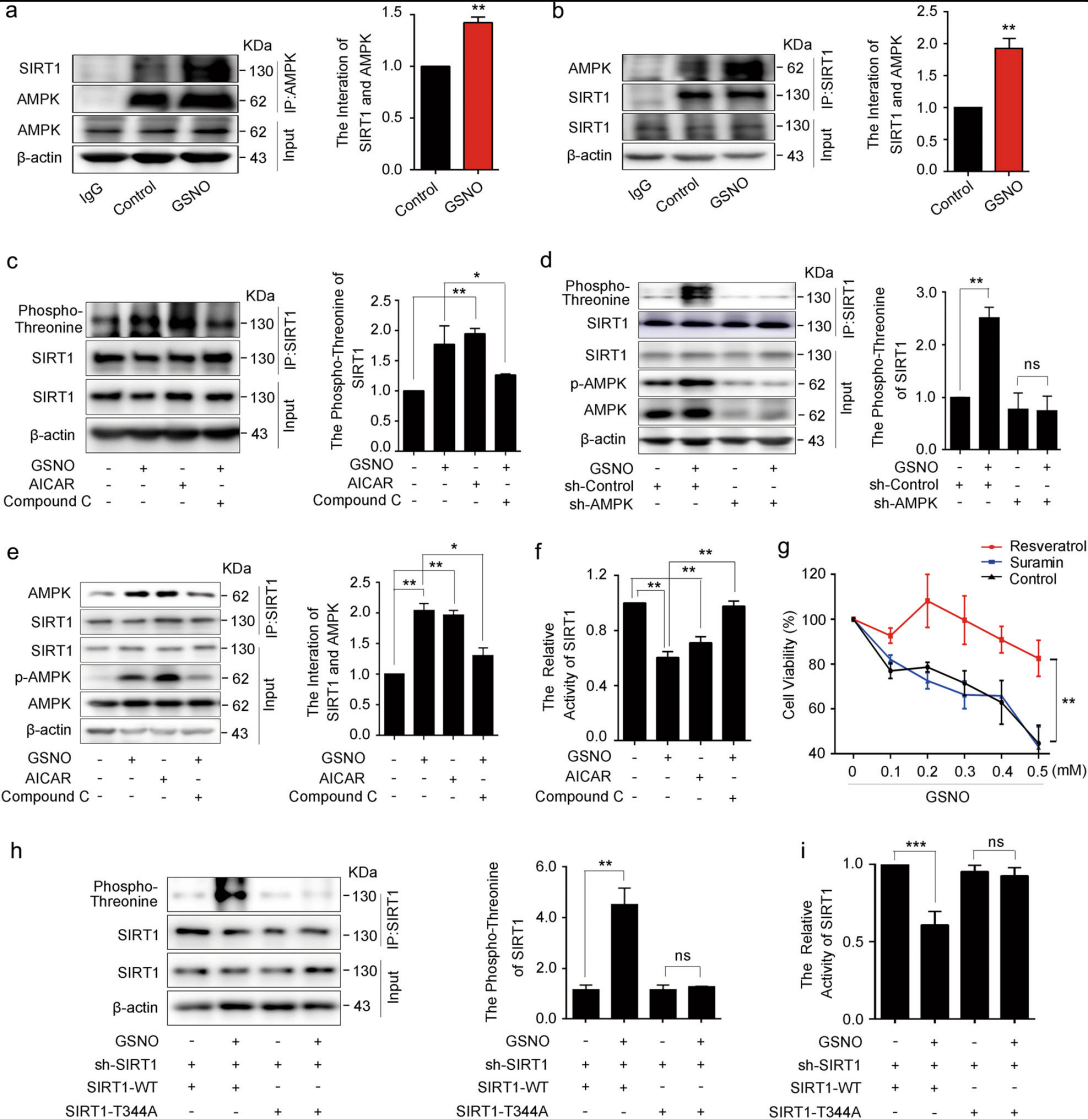
AMPK phosphorylation inhibits SIRT1 activity. **a**, **b** Co-immunoprecipitation of AMPK with SIRT1 in A549 cells (**P *<* 0.01, using unpaired two-tailed Student’s *t*-test). **c** SIRT1 was immunoprecipitated and analyzed for threonine phosphorylation with AICAR (1 mM) or Compound C (20 μM) (*P *<* 0.05, **P *<* 0.01, using one-way ANOVA followed by Bonferroni’s multiple comparisons test). **d** The effect of GSNO on threonine phosphorylation of SIRT1 in sh-Control and sh-AMPK A549 cells (**P *<* 0.01; ns, not significant, using unpaired two-tailed Student’s *t*-test). **e** Co-immunoprecipitation of AMPK with SIRT1 with AICAR or Compound C (*P *<* 0.05, **P *<* 0.01, using one-way ANOVA followed by Bonferroni’s multiple comparisons test). **f** The activity of SIRT1 with AICAR or Compound C (**P *<* 0.01, using one-way ANOVA followed by Bonferroni’s multiple comparisons test). **g** The effect of GSNO on cell viability with Resveratrol (50 μM) or Suramin (50 μM) (**P *<* 0.01, using two-way ANOVA followed by Bonferroni’s multiple comparisons test). **h** Threonine phosphorylation of SIRT1 in SIRT1 WT and T344A mutants (**P *<* 0.01; ns, not significant, using one-way ANOVA followed by Bonferroni’s multiple comparisons test). **i** The activity of SIRT1 in SIRT1 WT and T344A mutants (***P *<* 0.001; ns, not significant, using one-way ANOVA followed by Bonferroni’s multiple comparisons test). The data are expressed as the mean ± SD of three independent experiments

SIRT1 is a cellular protective regulator that could be regulated by AMPK^25^. Our results showed GSNO significantly increased the formation of AMPK/SIRT1 complex (Fig. 5a, b), suggesting that the interaction between AMPK and SIRT1 might be affected by GSNO. We further detected the phosphorylation of SIRT1. AICAR and GSNO treatment resulted in a dramatic phosphorylation of SIRT1 at threonine (Fig. 5c, Supplementary Fig. 6a), which was abolished by Compound C (Fig. 5c, Supplementary Fig. 6a), knockdown of AMPKα2 (Fig. 5d), or T172A-mutant AMPK expression (Supplementary Fig. 6b). Furthermore, GSNO or AICAR treatment enhanced association of SIRT1 with AMPK (Fig. 5e). To understand the effect of SIRT1 phosphorylation mediated by AMPK, SIRT1 activity assays were carried out. We found it was decreased in A549 and NCI-H1299 cells after GSNO or AICAR treatment, which was abolished by Compound C (Fig. 5f, Supplementary Fig. 6c). As a potent activator of SIRT1, resveratrol attenuated the cell damage caused by GSNO; however, SIRT1 deacetylase inhibitor suramin could not further contribute to cell damage in A549 cells (Fig. 5g, Supplementary Fig. 6d). In addition, SIRT1 overexpression attenuated the cell damage caused by GSNO (Supplementary Fig. 6e, f). It was reported that AMPK activation induced SIRT1 phosphorylation at Thr344 site, which attenuated the affinity of SIRT1 for p53 and lead to a loss of SIRT1 deacetylase activity toward p53^26^. To confirm whether GSNO-induced phosphorylation of SIRT1 is also at Thr344 site, non-phosphorylatable (T344A) mutants of SIRT1 was assessed for phosphorylation. The results revealed that treatment with GSNO significantly enhanced the wild-type SIRT1 threonine phosphorylation, but without any effect on the T344A-mutant SIRT1 (Fig. 5h), implying that AMPK activation induces the phosphorylation of SIRT1 at Thr344. Consistently, GSNO treatment inhibited the deacetylation activity of wild-type but not T344A-mutant SIRT1 (Fig. 5i). Moreover, adding H_2_O_2_ to A549 cells could mimic the effect of GSNO on the threonine phosphorylation of SIRT1, the association of SIRT1 and AMPK, and the deacetylation activity (Supplementary Fig. 6g–j). It is reported that *S*-nitrosylation inhibits the activity of the protein deacetylase SIRT1^27^. Therefore, we test the *S*-nitrosylation of SIRT1. The results showed that even though SIRT1 had *S*-nitrosylation expression in control group, treatment with GSNO for 24 h did not increase its level of *S*-nitrosylation (Supplementary Fig. 6k), implying that the decrease of SIRT1 activity induced by GSNO treatment is independent of SIRT1 *S*-nitrosylation.

The above results indicate that local H_2_O_2_ accumulation induced by GSNO increases AMPK activity, which further inhibits SIRT1 deacetylase activity via phosphorylating SIRT1 at Thr344 site.

### GSNO induces A549 apoptosis via p53-dependent p21 induction

**Fig. 6:**
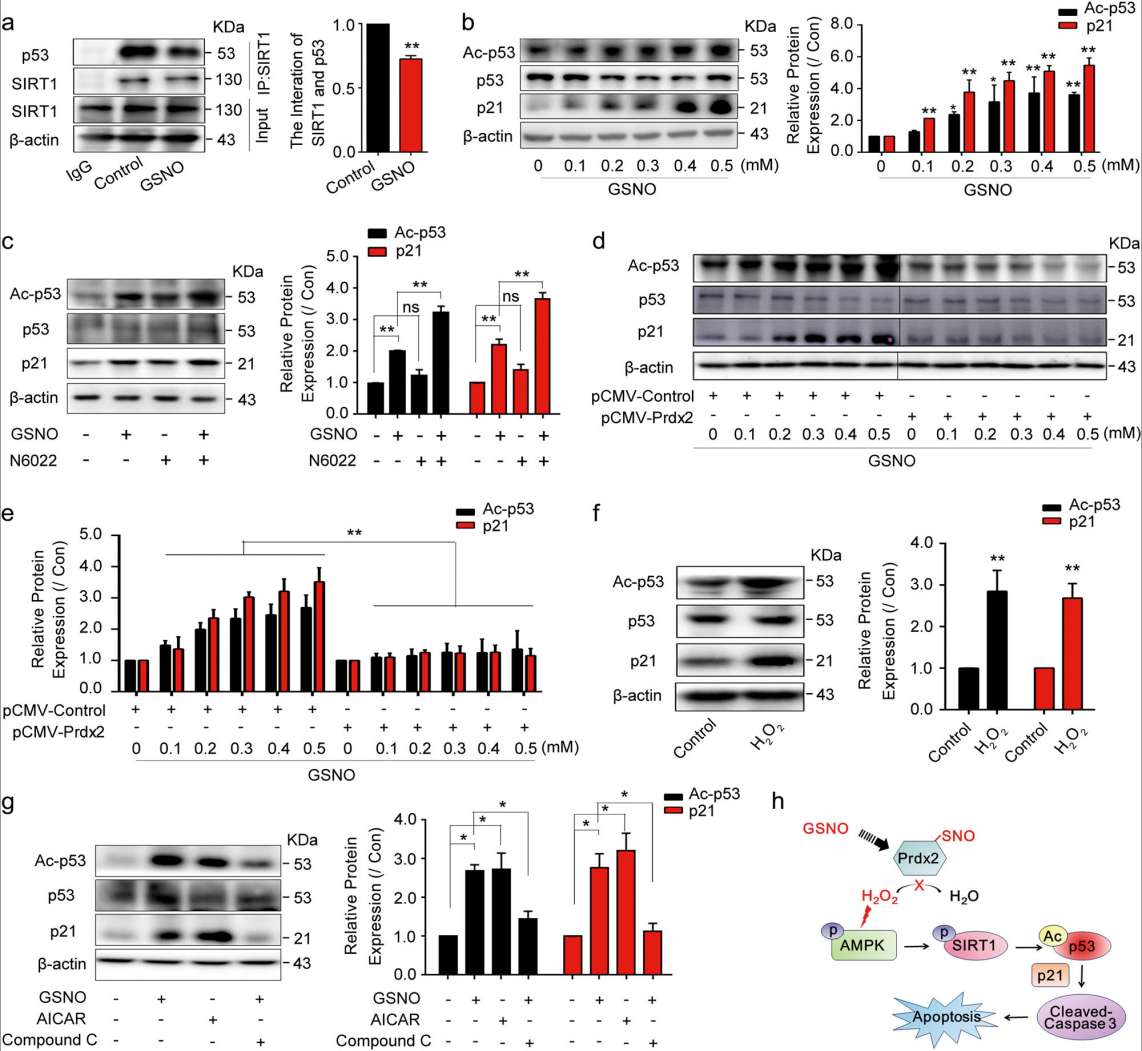
GSNO induces A549 apoptosis via p53-dependent p21 induction. **a** The interaction between SIRT1 and p53 (**P *<* 0.01, using unpaired two-tailed Student’s *t*-test). **b** Expression of p53 acetylation and p21 after GSNO treatment for 24 h (*P < 0.05, **P < 0.01, using one-way ANOVA followed by Bonferroni’s multiple comparisons test). **c** The effect of N6022 (0.5 μM) on the expression of p53 acetylation and p21 after GSNO treatment (**P *<* 0.01; ns, not significant, using one-way ANOVA followed by Bonferroni’s multiple comparisons test). **d**, **e** Overexpression Prdx2 attenuated the increased expression of p53 acetylation and p21 induced by GSNO in A549 cells (**P *<* 0.01, using two-way ANOVA followed by Bonferroni’s multiple comparisons test). **f** Expression of p53 acetylation and p21 after H_2_O_2_ (50 μM) treatment (**P *<* 0.01, using unpaired two-tailed Student’s *t*-test). **g** The effect of GSNO on the expression of p53 acetylation and p21 with AICAR (1 mM) or Compound C (20 μM) (*P *<* 0.05, using one-way ANOVA followed by Bonferroni’s multiple comparisons test). **h** Schematic representation of the mechanism by which GSNO promotes apoptosis in A549 cells. The data are expressed as the mean ± SD of three independent experiments

It is reported that SIRT1 deacetylates p53 and inhibits p53-mediated cell death following DNA damage^28^. Consistently, we found GSNO impaired the interaction between SIRT1 and p53 (Fig. 6a), and increased the acetylation of p53 (Fig. 6b). p21, as a major target of p53 activity, which links DNA damage to cell cycle arrest, was activated by GSNO (Fig. 6b). N6022 and LPS had synergistic effects with GSNO on the activation of p53 and p21 (Fig. 6c, Supplementary Fig. 7a). These effects induced by GSNO could be reversed by application of DTT or overexpression of Prdx2 (Fig. 6d, e, Supplementary Fig. 7b, c). Moreover, adding H_2_O_2_ to A549 cells could mimic the effect of GSNO on the p53 acetylation and the expression of p21 (Fig. 6f). In addition, GSNO or AICAR treatment increased p53 acetylation and the expression of p21, which was abolished by Compound C (Fig. 6g). These results suggest that GSNO decreases the deacetylase activity of SIRT1 toward p53 and increases p53 acetylation. Acetylated p53 increases the expression of p21 and further promotes apoptosis in A549 cells (Fig. 6h).

### GSNO induces H1299 apoptosis via FOXO1 activation

**Fig. 7:**
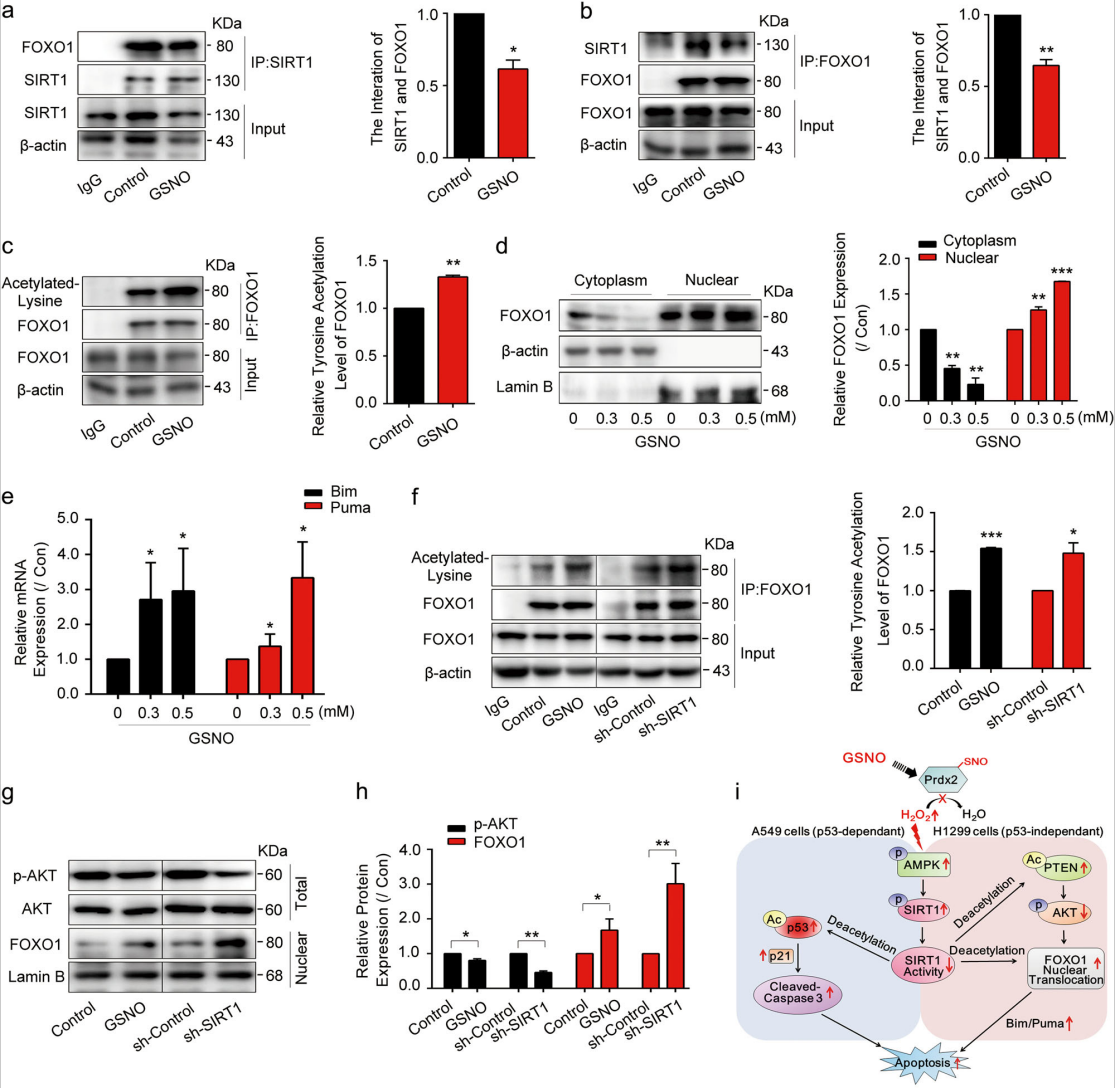
GSNO induces NCI-H1299 apoptosis via FOXO1 activation. **a**, **b** The interaction between SIRT1 and FOXO1 (*P *<* 0.05, **P *<* 0.01, using unpaired two-tailed Student’s *t*-test). **c** Detection of FOXO1 acetylation by immunoprecipitation (**P *<* 0.01, using unpaired two-tailed Student’s *t*-test). **d** FOXO1 expression in the cytoplasm and nucleus after GSNO treatment (**P < 0.01, ***P < 0.001, using one-way ANOVA followed by Bonferroni’s multiple comparisons test). **e** The mRNA quantification of *Bim* and *Puma* (*P < 0.05, using one-way ANOVA followed by Bonferroni’s multiple comparisons test). **f** Immunoprecipitation detection of FOXO1 acetylation with GSNO treatment or SIRT1 knockdown (*P *<* 0.05, ***P *<* 0.001, using unpaired two-tailed Student’s *t*-test). **g**, **h** The expression of FOXO1 and phosphorylated AKT with GSNO treatment or SIRT1 knockdown (*P *<* 0.05, **P *<* 0.01, using unpaired two-tailed Student’s *t*-test). **i** Schematic representation of the mechanism by which GSNO promotes apoptosis in lung cancer cells. The data are expressed as the mean ± SD of three independent experiments

Although the above results showed that GSNO induced A549 cell apoptosis via p53 pathway, it raised another question why p53-null NCI-H1299 cells were killed by GSNO. It was reported that SIRT1 regulates cellular protective and apoptotic processes by deacetylating some vital proteins, including p53, Ku70, phosphatase and tensin homolog (PTEN), and FOXO transcription factors^29,30^. Interestingly, we found GSNO decreased the interaction between SIRT1 and FOXO1 in NCl-H1299 cells (Fig. 7a, b), and the acetylation level of FOXO1 was significantly increased (Fig. 7c). Similarly, GSNO treatment also abrogated the binding of SIRT1 to PTEN (Supplementary Fig. 8a, b) and elevated the acetylation level of PTEN (Supplementary Fig. 8c). Experimental evidence indicates that the tumor suppressor activity of PTEN mostly relies on its counteracting activity on the phosphatidylinositol 3-kinase (PI3K)/protein kinase B (AKT) survival pathway, favoring apoptosis and/or cell cycle arrest^31^. The result showed that the phosphorylation of AKT decreased after GSNO treatment (Supplementary Fig. 8d). FOXO1 is a well-known target of AKT and the phosphorylation of FOXO1 by AKT causes FOXO1 nuclear exclusion and inactivation^32^. Consistently, we found that GSNO treatment increased the expression of nuclear FOXO1 (Fig. 7d). These results indicate that GSNO increased PTEN acetylation, inhibited AKT phosphorylation, and promoted FOXO1 nuclear translocation in p53-null NCI-H1299 cells. In line with the accumulation of FOXO1 in nuclear, the expression of its target genes *Bim* and *Puma* was significantly increased (Fig. 7e). However, in A549 cells, the acetylation of FOXO1 and PTEN was not obviously increased after GSNO treatment (Supplementary Fig. 9a–d). We suspected that GSNO induced cell apoptosis mainly via p53 in A549 cells. In NCI-H1299 cells, inhibiting FOXO1 or PTEN by adding AS1842856 or VO-Ohpic trihydrate rescued decreased cell viability caused by GSNO (Supplementary Fig. 9e, f). However, in A549 cells, p53 inhibitor Pifithrin-α, but not FOXO1 inhibitor AS1842856, could rescue GSNO-induced decrease of cell viability (Supplementary Fig. 9g, h). To confirm whether SIRT1 inactivation contributed to FOXO1 nuclear translocation, sh-SIRT1 was used in NCI-H1299 cells. The results showed silence of SIRT1 promoted the acetylation of FOXO1 and PTEN (Fig. 7f, Supplementary Fig. 8e), as well as FOXO1 nuclear translocation (Fig. 7g, h). We also focused on the possibility whether PTEN can be inhibited by nitrosylation. However, we did not observe any nitrosylation of PTEN when treated with GSNO (Supplementary Fig. 8f). Collectively, these data demonstrate GSNO induces the apoptosis in NCI-H1299 cells via FOXO1 activation (Fig. 7i).

## Discussion

Previous studies indicated that GSNO can induce apoptosis in various cells^11,33,34^ by the activation of many signaling pathways, including p53, nuclear factor-κB, and STAT3 pathways^35^. However, whether there are other pathways involved in it and the details of the signaling pathway still remain unclear. In this study, we reported that GSNO induced lung cancer cell apoptosis via Prdx2 and AMPK pathway.

Prdx2 has been found to be elevated in several human cancer tissues, including colorectal cancer^36^, and it affects diverse cellular processes involving cell proliferation and apoptosis via Wnt/β-catenin, transforming growth factor-β1, and PI3K/AKT^14,37,38^. Thus, inhibition of Prdx2 expression or activity is expected to be a new strategy for treating tumors. For example, Prdx2 knockdown inhibited colorectal cancer cell growth, which had high Prdx2 mRNA expression. However, Prdx2 mRNA level is low, whereas the protein expression level is high in the lung. Therefore, to regulate Prdx2 activity through posttranslational modification is an alternative way to inhibit lung cancer cell growth. Our findings suggest that it is possible to inhibit Prdx2 enzyme activity by nitrosylation.

There is a report that GSNO decreased the activation of AMPK, which was activated in cerebral ischemia reperfusion (IR) brains^39^. This seems to be opposite with our results and we guess there are two possible reasons. First, the state of AMPK is different: the AMPK has already been over activated in IR brains. Second, GSNO has different roles in oxidation system. In our lung cancer cellular models, it contributes H_2_O_2_ accumulation and promotes oxidative effects, whereas GSNO can also protect against oxidative stress in the endothelium, myocardium, brain tissues, and other cells^10^. The above two differences may explain the opposite effects of GSNO on AMPK.

Most importantly, we further revealed why AMPK activation induced by GSNO caused cell apoptosis. As previously reported, AMPK is involved in diverse protein phosphorylation^40^. For example, AMPK induced phosphorylation of SIRT1 at Thr344. It was reported that SIRT1 was phosphorylated and inactivated by AMPK at Thr344, promoting p53 acetylation and apoptosis of HCC cells^26^. In this study, GSNO significantly increased AMPK/SIRT1 interaction. Moreover, treatment with GSNO significantly enhanced the threonine phosphorylation of wild-type SIRT1 rather than T344A-mutant SIRT1, implying that AMPK activation induces the phosphorylation of SIRT1 at Thr344.

In summary, our study demonstrates that GSNO could induce lung cancer cell apoptosis via nitrosylating Prdx2 to induce H_2_O_2_ accumulation, subsequently increasing AMPK phosphorylation and further inhibiting the activity of SIRT1. SIRT1 inactivation induced apoptosis via p53 acetylation or FOXO1 activation in lung cancer cells with different p53 status. This discovery implies that GSNO could be a candidate therapeutic compound applicable to a wide range of human cancers regardless of their p53 status. Moreover, clinical data show that high expression of Prdx2 was closely correlated with unfavorable prognosis. Collectively, Prdx2 may be regarded as a potential target for clinical treatment of non-small cell lung cancer.

## Materials and methods

### Reagents and antibodies

Cell culture reagents were purchased from GE and fetal bovine serum (FBS) was purchased from PAN-Biotech; DTT was purchased from Millipore (Massachusetts, USA); Compound C, AICAR, Resveratrol, Suramin, SNAP, and ODQ were purchased from Sigma-Aldrich; N6022, Pifithrin-α, VO-Ohpic trihydrate, AS1842856, and MK 2206 were from MCE. Hydrogen peroxide (H_2_O_2_) was from Sinopharm Chemical Reagent; 3-(4,5-dimethyl-2-thiazolyl)−2,5-diphenyl-2-H-tetrazolium bromide (MTT) was purchased from Sangon Biotech; Lipofectamine® 3000 and Attractene were from Thermo Fisher Scientific and Qiagen, respectively. Fluor-de-Lys SIRT1 fluorometric drug discovery assay kit was from Enzo Life Sciences; Hydrogen Peroxide Assay Kit, Nitric Oxide Assay Kit, RIPA lysis buffer, Bicinchoninic Acid assay Kit, and Nuclear and Cytoplasmic Protein Extraction Kit were from Beyotime; Super ECL Prime was from US Everbright®Inc. The mRNA extraction kit was from Bioteke. Protein A Magnetic Beads and the Muse Count & Viability Assay Kit were purchased from Millipore. Anti-Flag M2 Magnetic Beads was from Sigma-Aldrich. Antibodies for phospho- and total-AMPK, acetyl- and total-p53, SIRT1, Cleaved- and total-Caspase-3, FOXO1, PTEN, β-actin, and acetylated-Lysine were from Cell Signaling Technology; antibodies for P21 was from Abcam; antibodies for Prdx2 was from Santa Cruz Biotechnology.

### GSNO synthesis

GSNO was synthesized as described previously^41^. In brief, the following reagents were prepared: glutathione (GSH) 400 mM, NaNO_2_ 400 mM, HCl 200 mM, and 40% NaOH. HCl (2 ml) was added to GSH (4 ml) for acidification and then NaNO_2_ (4 ml) was added for 20 min in ice bath. NaOH (40%) was added and pH adjusted to around 7.0 and characterized by UV spectroscopy.

### Cell culture treatment and transfection

NCI-H1299, A549, WI38, BEAS-2B, and 293T were obtained from the American Type Culture Collection (Manassas, VA, USA). Cell lines were grown in RPMI 1640 medium or Dulbecco’s modified Eagle’s medium supplemented with 10% FBS. Cell lines were grown at 37 °C in a humidified atmosphere containing 5% CO_2_ and 95% air. Cells were treated with different doses of GSNO for 24 h. Cells were transfected with different vectors using Attractene or Lipofectamine® 3000 according to the manufacturer’s protocol. The overexpression efficiency was shown in Supplementary Fig. 10a–d.

### Cell survival assay

The cells in 96-well culture plates were treated with different doses of GSNO for 24 h. MTT solution in media was added to each well at a final concentration of 1 mg/mL. The plate was incubated for 4 h at 37 °C. The medium was discarded, formazan crystals were dissolved in dimethyl sulfoxide, and the absorbance was measured at 490 nm. Experiments were performed in triplicate.

### Colony-formation assays

For colony-formation assays, A549 and NCI-H1299 cells were seeded in 6-well culture plates (400 cells/well) and grown for 10–14 days. Colonies were fixed with paraformaldehyde (4.0% v/v), stained with crystal violet (0.5% w/v), and photographed.

### Flow cytometry analysis

Muse Count & Viability reagent (20 μl) and then cell suspension (380 μl) were added to each tube. The tube was incubated for 5 min at room temperature and the cell count and viability were detected using Merck & Millipore Muse Cell Analyzer.

### Biotin-switch assay

Cells were lysed in appropriate amount of lysis buffer (50 mM NaCl, 25 mM Hepes, 0.1 mM EDTA, 0.1 mM neocuproine, 1% NP-40, 0.5 mM phenylmethylsulfonyl fluoride plus protease inhibitors, pH 7.4), unmodified protein –SH groups were blocked with 10% methyl-methanethiosulfonate for 30 min at 50 °C. Protein extracts were precipitated with cold acetone (at −20 °C) and resuspended in solubilizing buffer (100 mM Hepes, 1 mM EDTA, 0.1 mM neocuproine, 1% SDS). Ascorbic acid was added to remove nitrosyl groups. The biotinylated proteins were biotinylated with biotin-HPDP and then detected by immunoblotting with biotin antibody or pulled down with streptavidin magnetic beads.

### –SNO detection

Saville-Griess assay was used to detect –SNO of total protein and Prdx2 as previously reported^42^. For total protein, in brief, untreated and GSNO-treated cell lysates were prepared by precipitation in iced acetone followed by dissolution in incubation buffer. Mercury displacement of NO from SNO proteins was elicited by 10 min of incubation with 100 μM HgCl_2_. Nitrite levels were determined colorimetrically after reaction with 100 μl of working Griess reagent (1:1 mixture of 1% sulfanilamide and 0.1% *N*-(1-naphthyl)-ethylenediamine dihydrochloride) and quantified in a spectrophotometer at A540 relative to sodium nitrite standards. For Prdx2, A549 cells were transfected with pCMV-Prdx2 and then purified Prdx2 protein using anti-Flag M2 magnetic beads. SNO quantification of Prdx2 was detected in the same way.

### H_2_O_2_ content detection

Analyses were performed using hydrogen peroxide assay kit. In brief, after treatment, cells were collected and the lysis buffer solution supplied with the kit was added at a ratio of 150 μl per 1 × 10^6^ cells. Then the cells were centrifuged at 10,000 × *g* for 15 min at 4 °C and the supernatants were collected. Test tubes containing 50 μl supernatants and 100 μl test solutions were placed at room temperature for 30 min and measured immediately with a spectrophotometer at a wavelength of 560 nm. The concentration of H_2_O_2_ released was calculated from standard concentration curve with triplicate experiments.

### NO content detection

Analyses were performed using NO assay kit according to the manufacturer’s protocol. In brief, we used a commercial kit to quantify the level of NO according to the manufacturer’s protocol. Cell and Tissue Lysis Buffer for Nitric Oxide Assay was used to lyse cells. Lysed cells were centrifuged at 10,000 × *g* for 10 min to remove debris. Test tubes containing 50 μl supernatants, 50 μl Griess Reagent I, and 50 μl Griess Reagent II were measured with a spectrophotometer at a wavelength of 540 nm.

### Prdx2 dimer/monomer detection

As reported previously^43^, after GSNO treatment for 24 h, A549 cells were digested, centrifuged, and resuspended in 1 mL D-hank’s containing 100 mM *N*-ethyl maleimide (NEM) to preserve the Prdx2 redox state. After 20 min incubation at 37 °C, cells were pelleted and lysed in 400 μl nonreducing lysis buffer (100 mM Tris-HCl, pH 6.8, 10% (v/v) glycerol, 2% (w/v) SDS, 0.01% (w/v) bromophenol blue) containing 100 mM NEM and immediately frozen at 20 °C for immunoblotting detection.

### Knockdown of SIRT1, AMPK, and Prdx2 using shRNA

The pLKO.1-shRNA-SIRT1-lentiviral vector, pLKO.1-shRNA-AMPK-lentiviral vector, and pLKO.1-shRNA-Prdx2-lentiviral vector were constructed (pLKO.1, a lentiviral vector, Addgene), SIRT1 target sequence: 5′-TGAGGAGGTCAACTTCATC-3′; AMPKα2 target sequence: 5′-GCCATAAAGTGGCAGTTAA-3′; Prdx2 target sequence: 5′-GGAAGTACGTGGTCCTCTT-3′. HEK293T cells were co-transfected with lentiviral vector, psPAX2 and pMD2G vectors for virus production. Stable cell lines were obtained by lentiviral infection and selection with puromycin (1 μg/ml) or hygromycin B (200 μg/ml) for 2 weeks. The knockout efficiency was shown in Supplementary Fig. 10e.

### Co-immunoprecipitation

Untreated and GSNO-treated cells were washed once with phosphate-buffered saline and 500 μL NETN lysis buffer (20 mM Tris, pH 8.0, 100 mM NaCl, 1 mM EDTA, 0.5% NP-40) lysed for 20 min on ice. Cell lysates were then centrifuged at 12,000 r.p.m., 4 °C for 10 min. The soluble fraction was collected and then 50 μL supernatant liquid was left as input; the rest of supernatant liquid was immunoprecipitated overnight with anti-SIRT1 or anti-AMPK antibody at 4 °C and then with protein A magnetic beads for another 4 h. After that, the protein A magnetic beads were washed three times with NETN buffer. The beads were then boiled for 10 min in 1% SDS loading buffer for WB with the indicated antibodies.

### Measurement of SIRT1 activity

SIRT1 enzymatic activities were measured in A549 and NCI-H1299 using the commercially available SIRT1 Fluorometric Kit according to the manufacturer’s instructions.

### Real-time quantitative PCR

Total RNA was extracted from A549 or NCI-H1299 cells by RNA extraction kit. Then, cDNA was synthesized using M-MLV Reverse Transcriptase (Takara) according to the manufacturer’s instructions. Detection of mRNA levels was performed using a 7500 Real-Time PCR System (Applied Biosystems) and SYBR Green master mix (Roche).The forward and reverse primers were shown in Supplementary Table 1. Real-time quantitative PCR was performed in triplicate and the mRNA levels of target genes were normalized to glyceraldehyde 3-phosphate dehydrogenase.

### Western blotting

Cells were homogenized in RIPA lysis buffer, followed with centrifugation (10,000 r.p.m., 10 min). Total protein concentration in the supernatant was determined with Bicinchoninic Acid assay. Ten microliters of lysates was resolved by SDS-polyacrylamide gel electrophoresis, transferred onto polyvinylidene difluoride membrane, and probed for the specified antibody overnight at 4 °C. Secondary antibodies, conjugated with horseradish peroxidase were incubated at room temperature for 1 h. Proteins were visualized using ECL.

### Statistical analysis

Data were expressed as mean values ± SD. The statistical and plotting software package GraphPad Prism 5.0 (GraphPad Software, America) was used to perform unpaired two-tailed Student’s *t*-test, one-way analysis of variance (ANOVA), or two-way ANOVA followed by Bonferroni’s multiple comparisons test. The data of Prdxs mRNA expression in tumor and normal tissue was obtained from GEO (GSE19188). The patient data for survival analysis was obtained from the Cancer Genome Atlas database via the cBioPortal for Cancer Genomics. The survival rate after surgery was calculated using the Kaplan–Meier method by SPSS 17.0 software (SPSS, Chicago, USA).

## Supplementary information

Supplementary Figure

Supplementary table
