# Supplementary material for: S-nitrosylation of the Peroxiredoxin-2 promotes S-nitrosoglutathione-mediated lung cancer cells apoptosis via AMPK-SIRT1 pathway
Source: Cell Death Dis. 2019 Apr 15;10(5):329. doi: 10.1038/s41419-019-1561-x (PMC6465399; doi:10.1038/s41419-019-1561-x)
Supplement: Supplementary file 1 — Supplementary Figure [file 41419_2019_1561_MOESM1_ESM.pdf]

Supplementary materials for

S-nitrosylation of the Peroxiredoxin-2 promotes S-nitrosoglutathione-mediated lung cancer cells apoptosis via AMPK-SIRT1 pathway

Yihan Zhang[1, 2, 3], Changning Sun[1, 2, 3], Guokai Xiao[1, 2, 3], Hui Shan[1, 2, 3], Luyao Tang[1, 2, 3], Yujiao Yi[1, 2, 3], Wengong Yu[1, 2, 3,*], Yuchao Gu[1, 2, 3,*]

[1] Key Laboratory of Marine Drugs, Ministry of Education; School of Medicine and Pharmacy, Ocean University of China, 5 Yushan Road, Qingdao, 266003 China.

[2] Laboratory for Marine Drugs and Bioproducts of Qingdao National Laboratory for Marine Science and Technology, Qingdao, 266200 China.

[3] Key Laboratory of Glycoscience & Glycotechnology of Shandong Province, Qingdao, 266003 China.

Supplementary Figure 1. GSNO induces lung cancer cell NCI-H1299 apoptosis.

(**a**) NCI-H1299 cells were treated with different concentrations of GSNO (0-0.5 mM) (Bar = 20 μm). (**b**) Cell viability analysis of NCI-H1299 cells after GSNO treatment. (**c**) Flow cytometry analysis of live cells after treating with GSNO. (**d**) Expression of Cleavage of Caspase-3 in NCI-H1299 cells was probed by western blot. (**e**) Colony formation of NCI-H1299 cells treated with GSNO. (**f**) The synergistic effects of GSNO (0.3 mM) with cisplatin (10 μM) or paclitaxel (20 μM) on NCI-H1299 cells. The data are expressed as the mean ± SD of three independent experiments. *P < 0.05, **P < 0.01, ***P < 0.001, using one-way ANOVA followed by Bonferroni's multiple comparisons test.

Supplementary Figure 2. SNAP or LPS induces A549 cell apoptosis.

(**a**) Cell viability analysis of lung cancer cells (A549 and NCI-H1299) and normal cells (WI38 and BEAS-2B) treated with GSNO for 24 h (***P < 0.001, using two-way ANOVA followed by Bonferroni's multiple comparisons test). (**b**) Cell viability analysis of A549 treated with GSNO (0.3 mM) or/and SNAP (200 μM) for 24 h (*P < 0.05, using one-way ANOVA followed by Bonferroni's multiple comparisons test). (**c**) Induction of Cleavage of Caspase-3 in A549 cells treated with GSNO or/and SNAP for 24 h (*P < 0.05, ***P < 0.001, using one-way ANOVA followed by Bonferroni's multiple comparisons test). (**d**) The NO production in A549 cells after SNAP or GSNO treatment (*P < 0.05, **P < 0.01, ***P < 0.001, using one-way ANOVA followed by Bonferroni's multiple

comparisons test). (**e**) Cell viability analysis of A549 treated with SNAP compared with GSNO (***P < 0.001, using two-way ANOVA followed by Bonferroni's multiple comparisons test). (**f**) The NO production in A549 cells after LPS treatment (**P < 0.01, ***P < 0.001, using one-way ANOVA followed by Bonferroni's multiple comparisons test). (**g**) Cell viability analysis of A549 treated with LPS for 24 h (*P < 0.05, **P < 0.01, ***P < 0.001, using one-way ANOVA followed by Bonferroni's multiple comparisons test). (**h**) Induction of Cleavage of Caspase-3 in A549 cells treated with GSNO or/and LPS (150 μg/ml) for 24 h (*P < 0.05, using one-way ANOVA followed by Bonferroni's multiple comparisons test). The data are expressed as the mean ± SD of three independent experiments.

Supplementary Figure 3. GSNO nitrosylates Prdx2 in NCI-H1299 cells.

(**a**) Effect of GSNO on cell number with or without ODQ (25 μM) for 24 h in A549 cells (ns, not significant, using two-way ANOVA followed by Bonferroni's multiple comparisons test). (**b**) Effect of GSNO on cell number with or without DTT (0.5 mM) for 24 h in A549 cells (***P < 0.001, using two-way ANOVA followed by Bonferroni's multiple comparisons test). (**c**) Effect of GSNO on cell viability with or without DTT (0.5 mM) for 24 h in NCI-H1299 cells (**P < 0.01, using two-way ANOVA followed by Bonferroni's multiple comparisons test). (**d**-**h**) mRNA expression level of Prdx1, Prdx3, Prdx4, Prdx5, Prdx6 in different kinds of human lung cancer tissues and normal tissues (*P < 0.05, ***P < 0.001, using one-way ANOVA followed by Bonferroni's multiple comparisons

test). (**i**, **j**) mRNA expression level of Prdx2 and Prdx4 in human lung cancer cells (A549 and NCI-H1299) and normal lung cells (WI38 and BEAS-2B, *$P < 0.05$, **$P < 0.01$, ***$P < 0.001$, ns, not significant, using one-way ANOVA followed by Bonferroni's multiple comparisons test). (**k**) Prdx2 protein expression in human lung cancer cells (A549 and NCI-H1299) and normal lung cells (WI38 and BEAS-2B) (*$P < 0.05$, **$P < 0.01$, ***$P < 0.001$, using one-way ANOVA followed by Bonferroni's multiple comparisons test). (**l**) Detection of Prdx2 nitrosylation by biotin-switch assay in NCI-H1299 cells (*$P < 0.05$, **$P < 0.01$, ns, not significant, using one-way ANOVA followed by Bonferroni's multiple comparisons test). (**m**) Detection for the nitrosylation of total protein and Prdx2 by saville-Griess assay (*$P < 0.05$, **$P < 0.01$, using unpaired two-tailed Student's *t*-test). (**n**) The cell viability of A549 cells transfected with pCMV-Prdx2 was detected by MTT assay (**$P < 0.01$, using two-way ANOVA followed by Bonferroni's multiple comparisons test). The data are expressed as the mean ± SD of three independent experiments.

Supplementary Figure 4. GSNO induces apoptosis in lung cancer cells via H_2O_2 accumulation.

(**a**) Detection of intracellular H_2O_2 after GSNO treatment for 24 h in NCI-H1299 cells (*$P < 0.05$, ***$P < 0.001$, using one-way ANOVA followed by Bonferroni's multiple comparisons test). (**b**) The effect of H_2O_2 (50 μM) on A549 cells (Bar = 20 μm). (**c**) Flow cytometry analysis of live A549 cells after treating A549 cells with H_2O_2 (50 μM). (**d**) Cell number analysis of A549 cells cultured in the

presence of H_2O_2 for 24 h (**P < 0.01, using unpaired two-tailed Student's t-test). (**e**) The effect of H_2O_2 (50 μM) on NCI-H1299 cells (Bar = 20 μm). (**f**) Flow cytometry analysis of live NCI-H1299 cells after treating with H_2O_2 (50 μM). (**g**, **h**) Cell viability and number analysis of NCI-H1299 cells cultured in the presence of H_2O_2 (50 μM) for 24 h (**P < 0.01, using unpaired two-tailed Student's t-test). The data are expressed as the mean ± SD of three independent experiments.

Supplementary Figure 5. GSNO induces cell death via activating the phosphorylation of AMPK.

(**a**) Expression of AMPK phosphorylation after GSNO treatment in NCI-H1299 cells (*P < 0.05, **P < 0.01, ***P < 0.001, using one-way ANOVA followed by Bonferroni's multiple comparisons test). (**b**) Expression of p-ACC and p-AMPK/AMPK in A549 cells treated with GSNO (0.3 mM) or/and SNAP (200 μM) (*P < 0.05, **P < 0.01, using one-way ANOVA followed by Bonferroni's multiple comparisons test). (**c**) Expression of p-ACC and p-AMPK/AMPK in A549 cells treated with GSNO or/and LPS (150 μg/ml) (*P < 0.05, **P < 0.01, using one-way ANOVA followed by Bonferroni's multiple comparisons test). (**d**) Expression of AMPK phosphorylation after H_2O_2 (50 μM) treatment in NCI-H1299 cells (**P < 0.01, using unpaired two-tailed Student's t-test). (**e**, **f**) The effect of AICAR (1 mM) or Compound C (20 μM) on AMPK phosphorylation in GSNO-treated A549 cells (***P < 0.001, using one-way ANOVA followed by Bonferroni's multiple comparisons test). (**g**) The effect of

GSNO on cell viability with AICAR or Compound C in NCI-H1299 cells (*P < 0.05, **P < 0.01, using two-way ANOVA followed by Bonferroni's multiple comparisons test). (**h**, **i**) The effect of GSNO on p-ACC and p-AMPK/AMPK expression in AMPK-WT and AMPK-T172A mutant groups (**P < 0.01, using two-way ANOVA followed by Bonferroni's multiple comparisons test). (**j**) The effect of GSNO on Cleaved-Caspase 3 expression in AMPK-WT and AMPK-T172A mutant groups (**P < 0.01, using two-way ANOVA followed by Bonferroni's multiple comparisons test). The data are expressed as the mean ± SD of three independent experiments.

Supplementary Figure 6. The activation of AMPK phosphorylates SIRT1 to inhibit its activity.

(**a**) SIRT1 was immunoprecipitated and analyzed for threonine phosphorylation with AICAR (1 mM) or Compound C (20 μM) in NCI-H1299 (*P < 0.05, **P < 0.01, using one-way ANOVA followed by Bonferroni's multiple comparisons test). (**b**) The threonine phosphorylation of SIRT1 in AMPK-WT and AMPK-T172A mutant groups (***P < 0.001, ns, not significant, using unpaired two-tailed Student's t-test). (**c**) The effect of GSNO on the activity of SIRT1 in NCI-H1299 cells (*P < 0.05, **P < 0.01, using one-way ANOVA followed by Bonferroni's multiple comparisons test). (**d**) Detection for the activity of SIRT1 after resveratrol (50 μM) or suramin (50 μM) treatment (**P < 0.01, using one-way ANOVA followed by Bonferroni's multiple comparisons test). (**e**, **f**) The effect of SIRT1 overexpression on cell viability in A549 cells (**e**) and

NCI-H1299 cells (**f**) after GSNO treatment (**P < 0.01, ***P < 0.001, using two-way ANOVA followed by Bonferroni's multiple comparisons test). (**g**, **h**) Threonine phosphorylation of SIRT1 after H_2O_2 treatment in A549 cells (*P < 0.05, using unpaired two-tailed Student's t-test). (**i**) Co-immunoprecipitation of AMPK with SIRT1 after H_2O_2 (50 μM) treatment in A549 cells (**P < 0.01, using unpaired two-tailed Student's t-test). (**j**) The activity of SIRT1 after H_2O_2 treatment in A549 cells (**P < 0.01, using unpaired two-tailed Student's t-test). (**k**) Detection of SIRT1 nitrosylation by biotin-switch assay. The data are expressed as the mean ± SD of three independent experiments.

Supplementary Figure 7. GSNO induces A549 cells apoptosis via p53-dependent p21 induction.

(**a**) Expression of p53 acetylation and p21 after GSNO (0.3 mM) or/and LPS (150 μg/ml) treatment (*P < 0.05, **P < 0.01, using one-way ANOVA followed by Bonferroni's multiple comparisons test). (**b**, **c**) DTT attenuated the increased expression of p53 acetylation and p21 induced by GSNO in A549 cells (**P < 0.01, using two-way ANOVA followed by Bonferroni's multiple comparisons test). The data are expressed as the mean ± SD of three independent experiments.

Supplementary Figure 8. GSNO induces NCI-H1299 apoptosis via FOXO1 activation.

(**a**, **b**) The interaction between SIRT1 and PTEN after treating NCI-H1299 cells

with GSNO (*$P < 0.05$, **$P < 0.01$, using unpaired two-tailed Student's *t*-test). (**c**) Detection of PTEN acetylation by immunoprecipitation after treating NCI-H1299 cells with GSNO (**$P < 0.01$, using unpaired two-tailed Student's *t*-test). (**d**) Expression of AKT phosphorylation after GSNO treatment (*$P < 0.05$, **$P < 0.01$, ***$P < 0.001$, using one-way ANOVA followed by Bonferroni's multiple comparisons test). (**e**) Detection of PTEN acetylation by immunoprecipitation in GSNO treatment or SIRT1 knockdown groups (*$P < 0.05$, **$P < 0.01$, using unpaired two-tailed Student's *t*-test). (**f**) Detection of PTEN nitrosylation by biotin-switch assay in indicated groups. The data are expressed as the mean ± SD of three independent experiments.

Supplementary Figure 9. GSNO induces A549 cells apoptosis independent of FOXO1 activation.

(**a**) The interaction between SIRT1 and FOXO1 after treating A549 with GSNO (ns, not significant, using unpaired two-tailed Student's *t*-test). (**b**) Detection of FOXO1 acetylation by immunoprecipitation after treating A549 with GSNO (ns, not significant, using unpaired two-tailed Student's *t*-test). (**c**) The interaction of SIRT1 with PTEN after treating A549 with GSNO (ns, not significant, using unpaired two-tailed Student's *t*-test). (**d**) Detection of PTEN acetylation by immunoprecipitation after treating A549 with GSNO (ns, not significant, using unpaired two-tailed Student's *t*-test). (**e**) The effect of GSNO on cell viability when VO-Ohpic trihydrate (50 nM), AS1842856 (0.5 μM) or MK2206 (10 μM) was added to NCI-H1299 cells (***$P < 0.001$, using two-way ANOVA followed

by Bonferroni's multiple comparisons test). (**f**) Induction of Cleavage of Caspase-3 in NCI-H1299 cells treated with GSNO or/and AS1842856 (0.5 μM) (**P* < 0.05, using one-way ANOVA followed by Bonferroni's multiple comparisons test). (**g**) The effect of GSNO (0.5 mM) on cell viability when Pifithrin-β (10 μM) or AS1842856 (0.5 μM) was added to A549 cells (***P* < 0.01, ns, not significant, using two-way ANOVA followed by Bonferroni's multiple comparisons test). (**h**) Induction of Cleavage of Caspase-3 in A549 cells treated with GSNO (0.5 mM) or/and Pifithrin-β (10 μM) (***P* < 0.01, using one-way ANOVA followed by Bonferroni's multiple comparisons test). The data are expressed as the mean ± SD of three independent experiments.

Supplementary Figure 10. The efficiency of overexpression for Prdx2 or SIRT1 and knockdown for SIRT1 or AMPKα2.

(**a**) Representative fluorescence images for transfection in lung cancer cells (Bar = 20 μm). (**b-d**) SIRT1 and Prdx2 expression levels were detected using western blot after cells were transfected with pCDH-SIRT1 or pCMV-Prdx2 plasmids (****P* < 0.001, using unpaired two-tailed Student’s *t*-test). (**e**) SIRT1 and AMPKα2 expression levels were detected after cells were transfected with sh-RNA (****P* < 0.001, using unpaired two-tailed Student’s *t*-test). The data are expressed as the mean ± SD of three independent experiments.

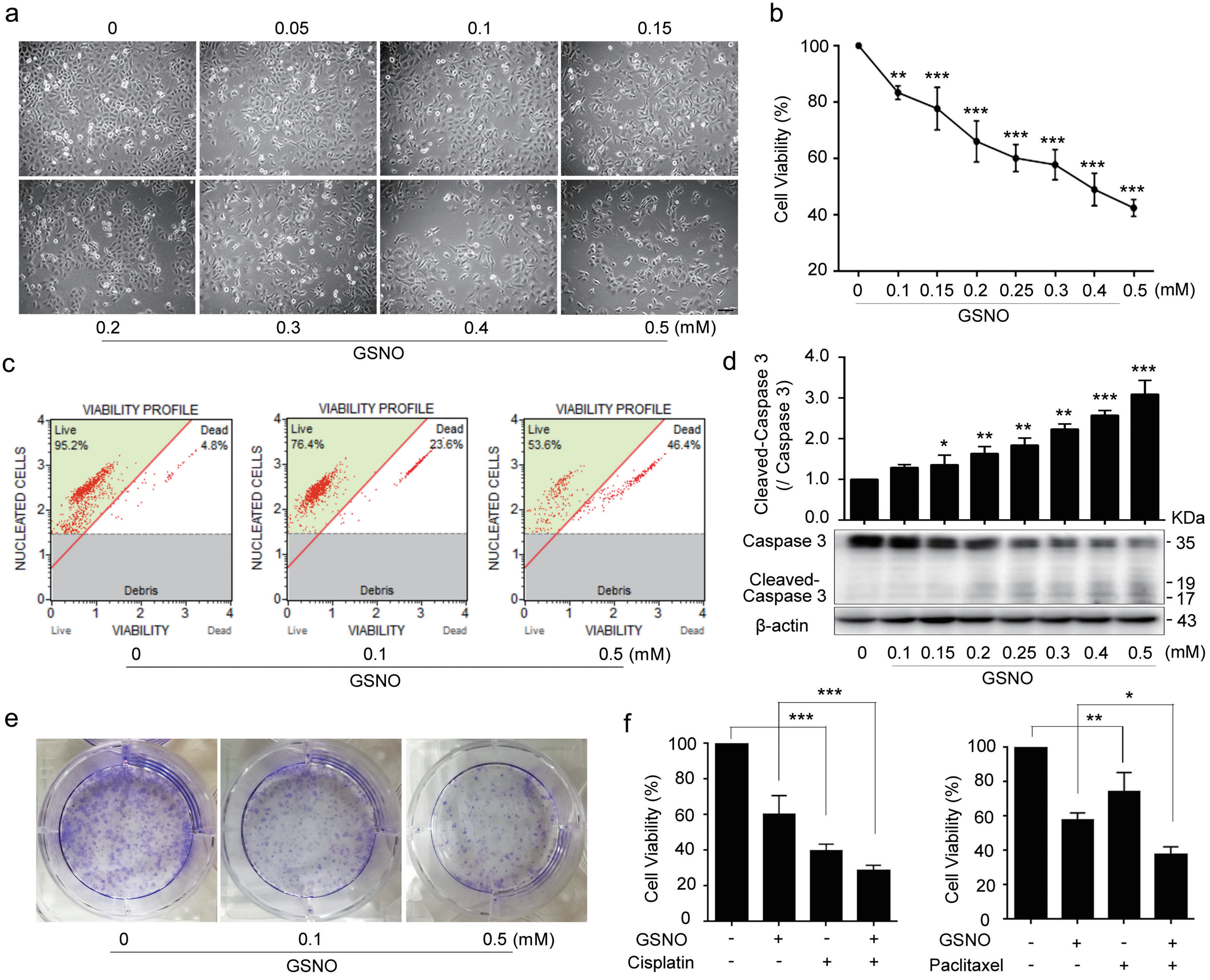

Supplementary Figure 1

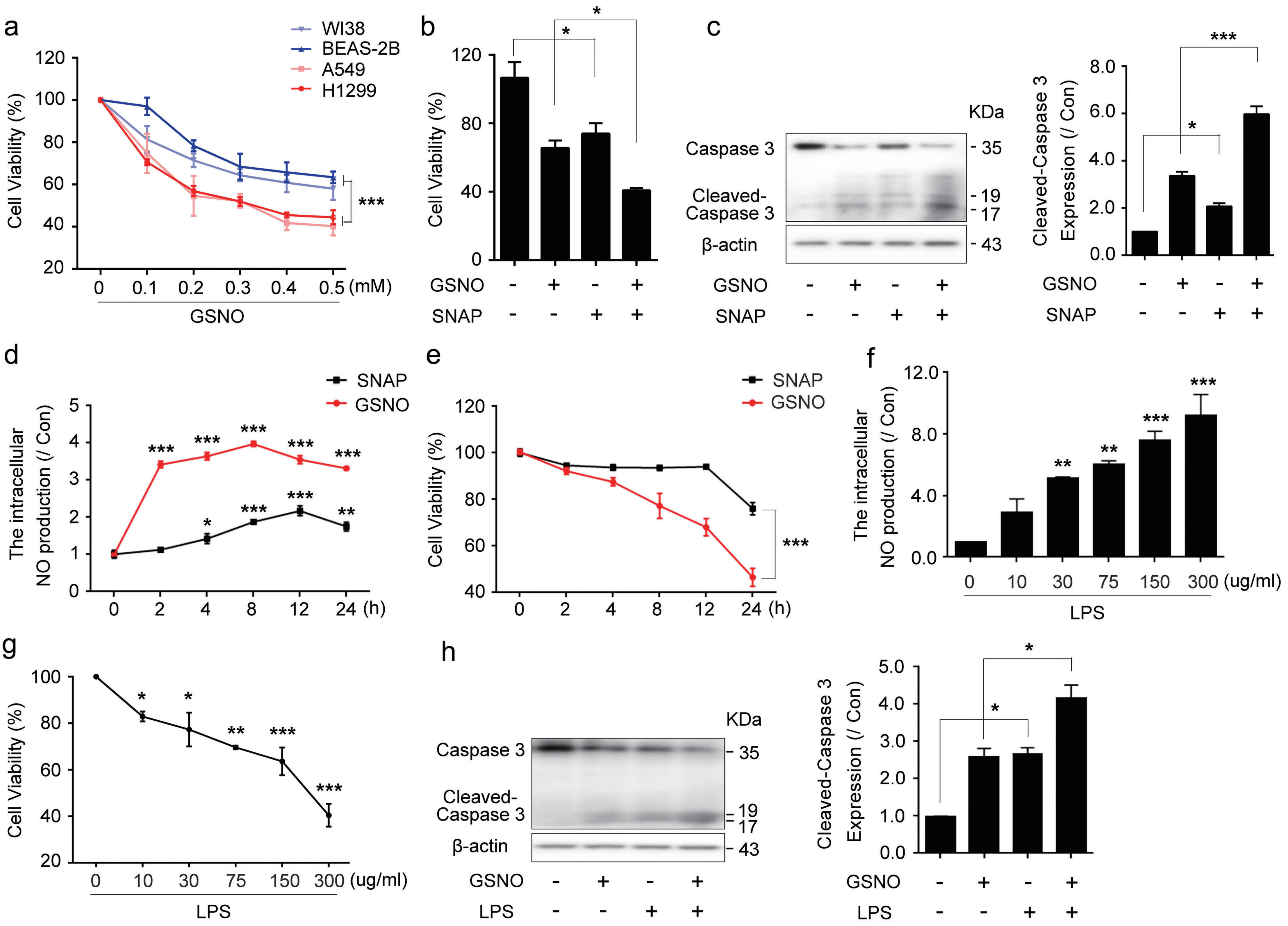

Supplementary Figure 2

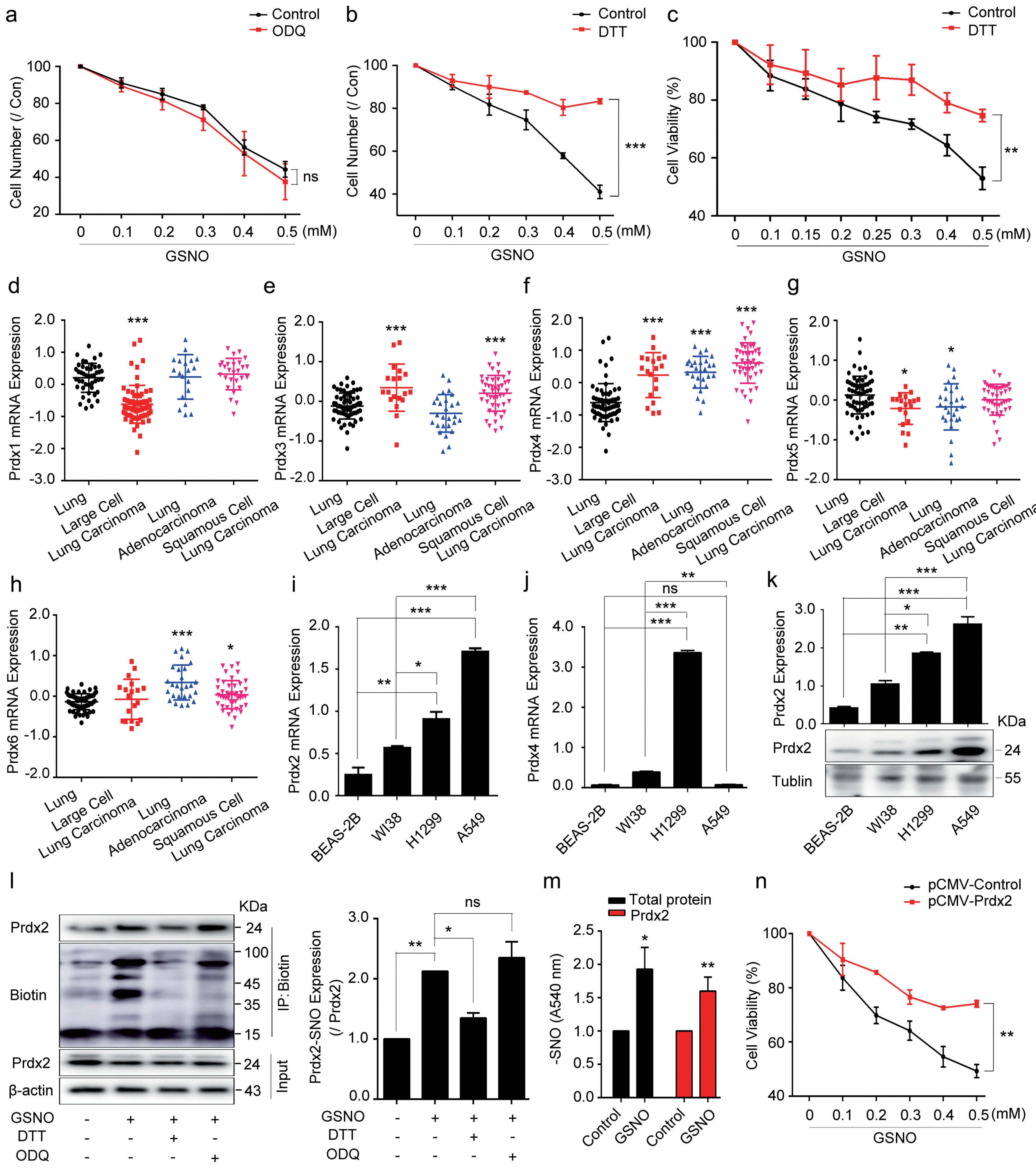

Supplementary Figure 3

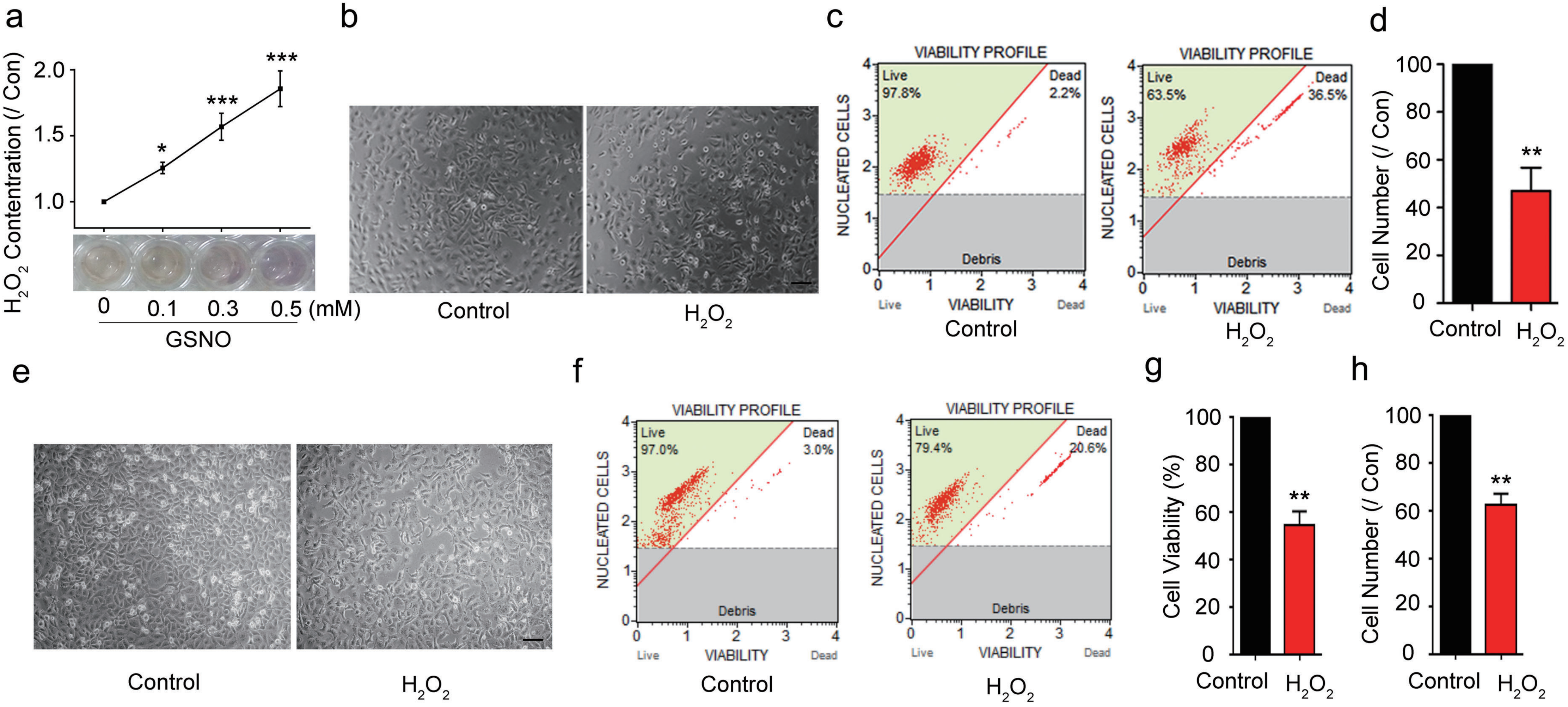

Supplementary Figure 4

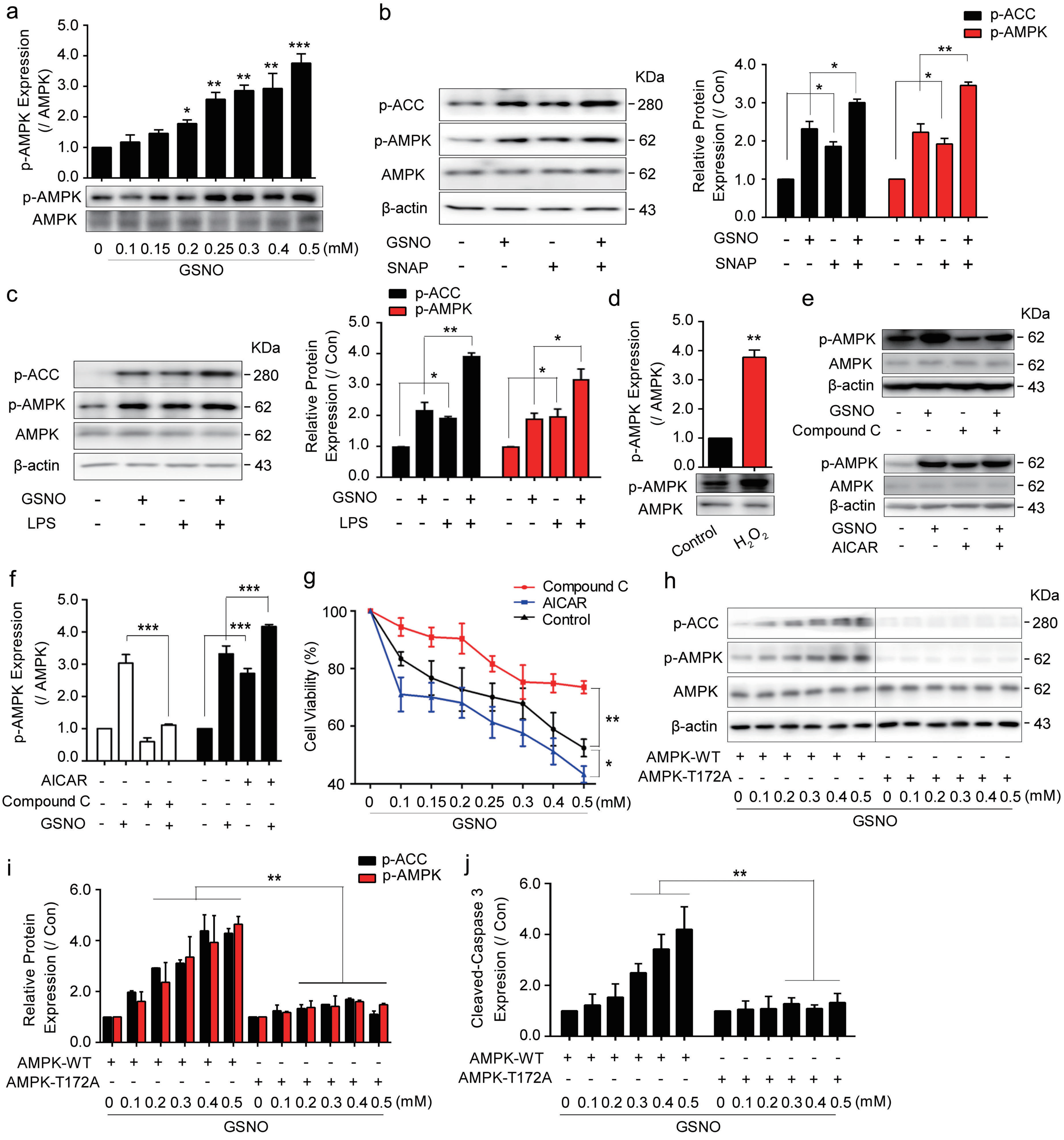

Supplementary Figure 5

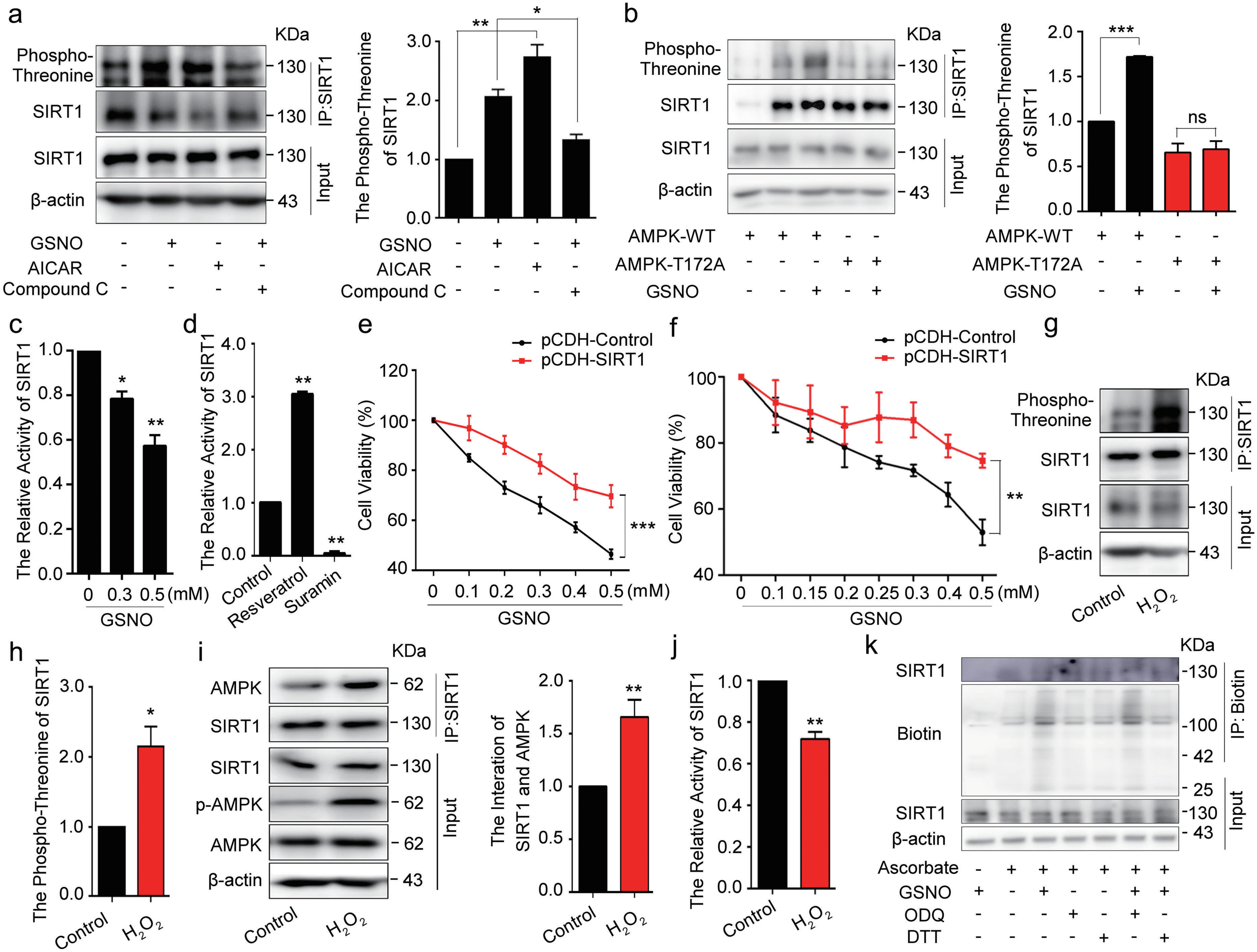

Supplementary Figure 6

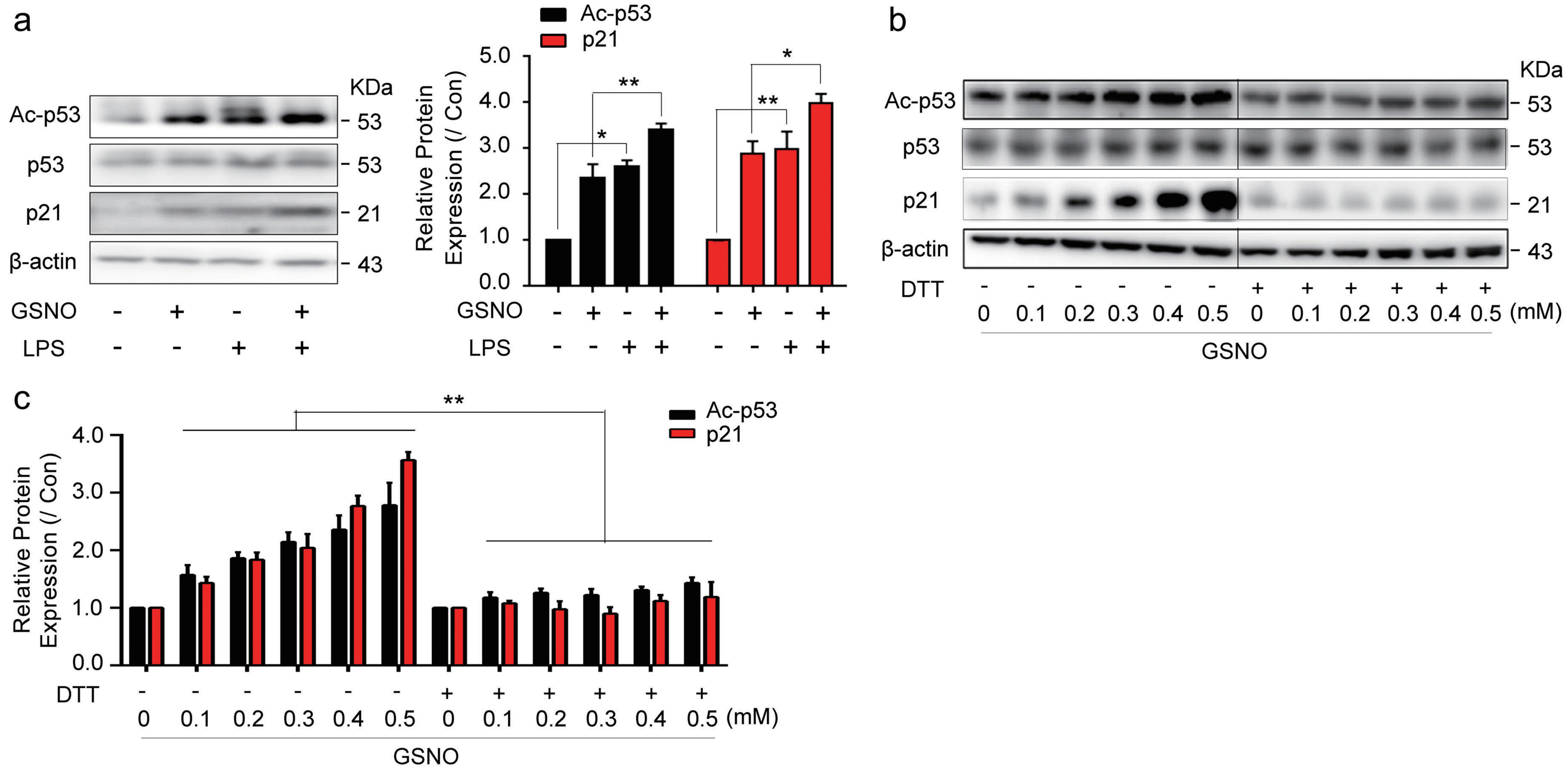

Supplementary Figure 7

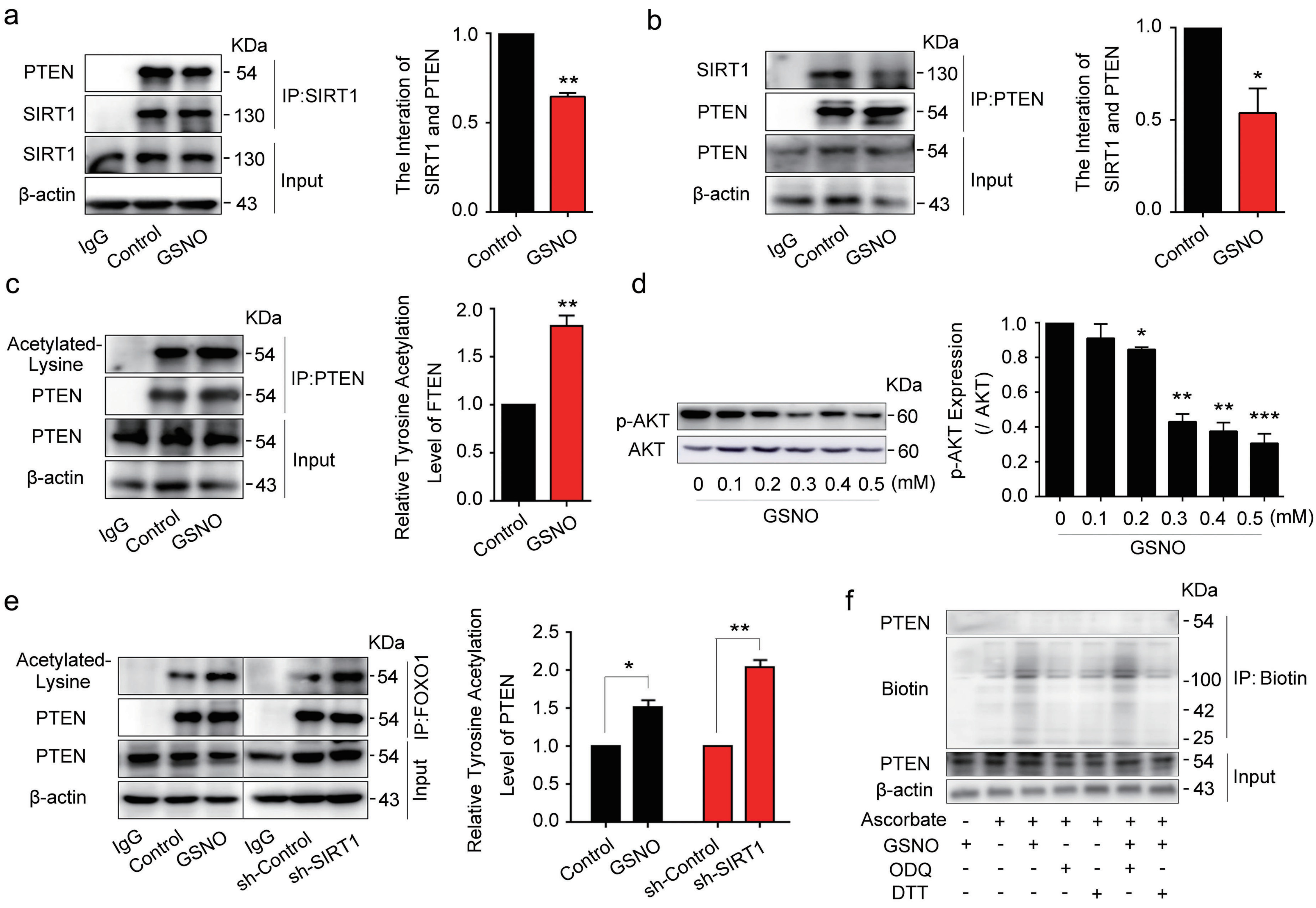

Supplementary Figure 8

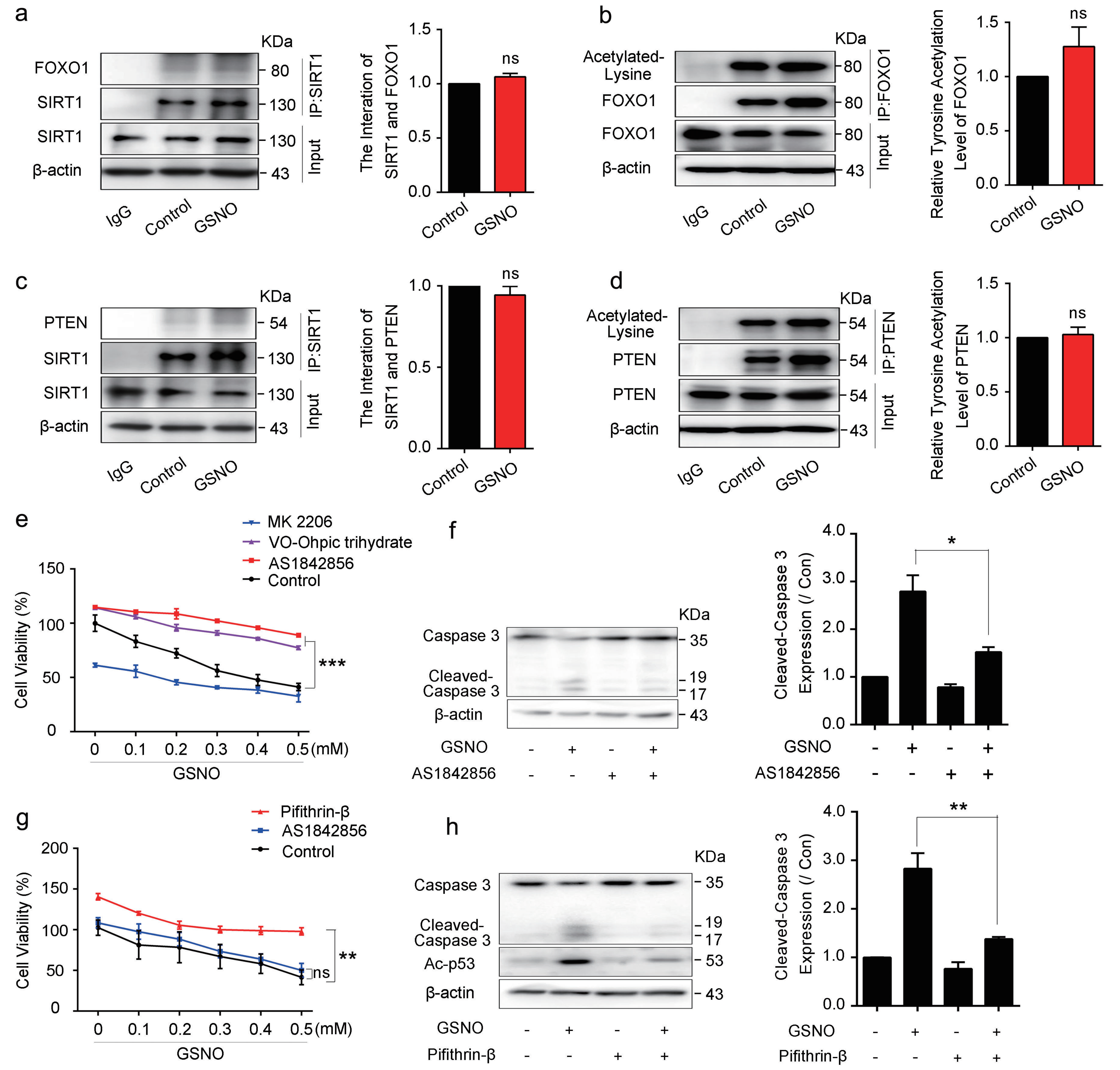

Supplementary Figure 9

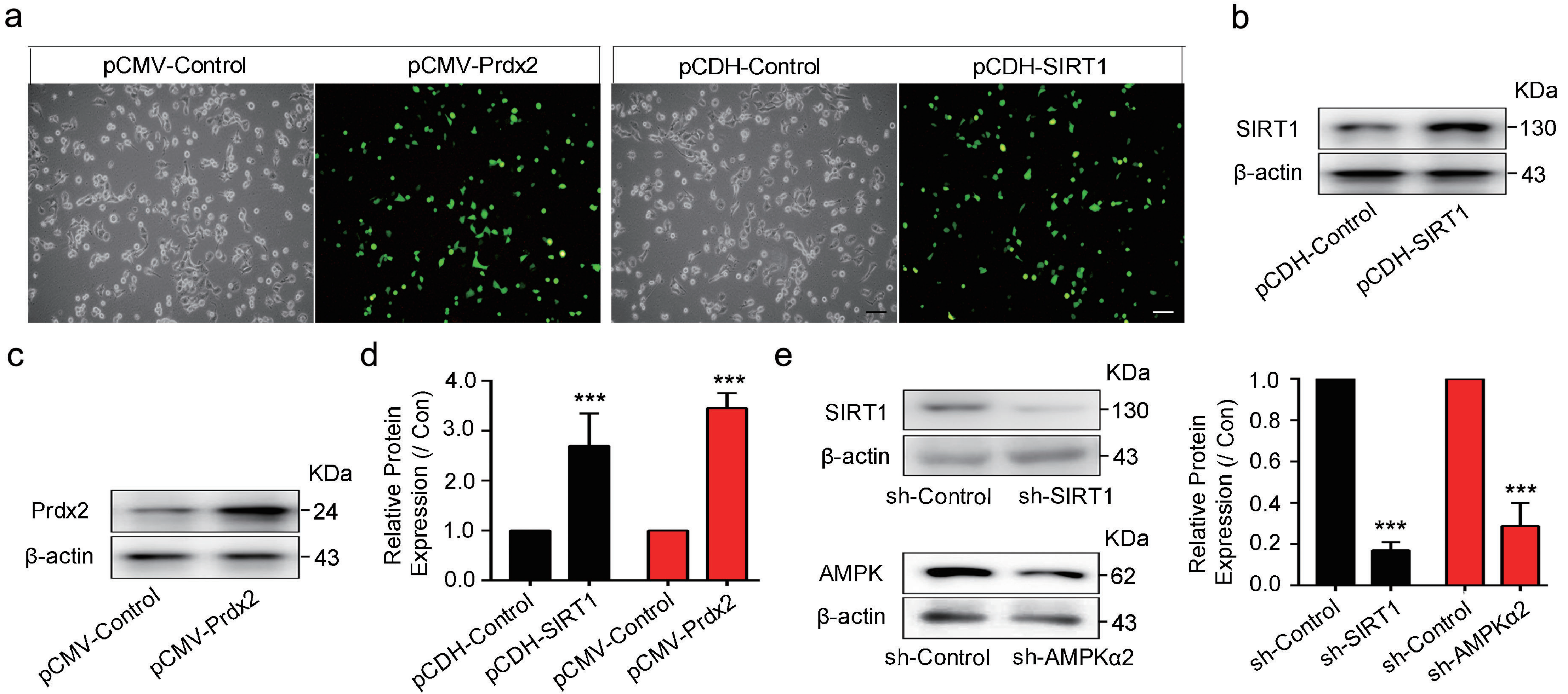

Supplementary Figure 10
