# Supplementary material for: S-nitrosylation of the Peroxiredoxin-2 promotes S-nitrosoglutathione-mediated lung cancer cells apoptosis via AMPK-SIRT1 pathway
Source: Cell Death Dis. 2019 Apr 15;10(5):329. doi: 10.1038/s41419-019-1561-x (PMC6465399; doi:10.1038/s41419-019-1561-x)
Supplement: Supplementary file 2 — Supplementary table [file 41419_2019_1561_MOESM2_ESM.docx]

**Supplementary Table 1.**

| qPCR primers | Sequence |
| --- | --- |
| Puma F | ATGGCGGACGACCTCAAC |
| Puma R | AGTCCCATGAAGAGATTGTACATGAC |
| Bim F | TATGGAGAAGGCATTGAC |
| Bim R | TGTGGTGATGAACAGAGG |
| GAPDH F | GCCTTCCGTGTTCCTACCC |
| GAPDH R | TGCCTGCTTCACCACCTTC |
